# PD-1 and ICOS coexpression identifies tumor-reactive CD4^+^ T cells in human solid tumors

**DOI:** 10.1172/JCI156821

**Published:** 2022-06-15

**Authors:** Rebekka Duhen, Olivier Fesneau, Kimberly A. Samson, Alexandra K. Frye, Michael Beymer, Venkatesh Rajamanickam, David Ross, Eric Tran, Brady Bernard, Andrew D. Weinberg, Thomas Duhen

**Affiliations:** 1 Earle A. Chiles Research Institute, Providence Cancer Institute, Portland, Oregon, USA.; 2 AgonOx Inc., Portland, Oregon, USA.

**Keywords:** Immunology, Oncology, Cancer, Immunotherapy, T cells

## Abstract

CD4^+^ Th cells play a key role in orchestrating immune responses, but the identity of the CD4^+^ Th cells involved in the antitumor immune response remains to be defined. We analyzed the immune cell infiltrates of head and neck squamous cell carcinoma and colorectal cancers and identified a subset of CD4^+^ Th cells distinct from FOXP3^+^ Tregs that coexpressed programmed cell death 1 (PD-1) and ICOS. These tumor-infiltrating lymphocyte CD4^+^ Th cells (CD4^+^ Th TILs) had a tissue-resident memory phenotype, were present in MHC class II–rich areas, and proliferated in the tumor, suggesting local antigen recognition. The T cell receptor repertoire of the PD-1^+^ICOS^+^ CD4^+^ Th TILs was oligoclonal, with T cell clones expanded in the tumor, but present at low frequencies in the periphery. Finally, these PD-1^+^ICOS^+^ CD4^+^ Th TILs were shown to recognize both tumor-associated antigens and tumor-specific neoantigens. Our findings provide an approach for isolating tumor-reactive CD4^+^ Th TILs directly ex vivo that will help define their role in the antitumor immune response and potentially improve future adoptive T cell therapy approaches.

## Graphical abstract

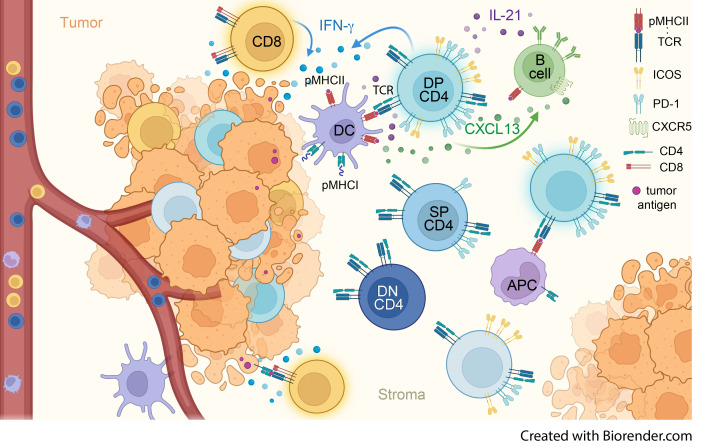

## Introduction

The realization that the immune system can recognize and kill cancer cells led to a paradigm shift in the therapeutic approach for patients with cancer. Instead of directly targeting the tumor, new therapies aim to unleash an ongoing antitumor immune response or induce an antitumor immune response when it is absent. The potential of immunotherapy for solid tumors is confirmed by the approval of therapies targeting the programmed cell death 1/programmed death ligand 1 (PD-1/PD-L1) pathway for patients with recurrent/metastatic melanoma or mismatch repair–deficient cancers, among others (1–3). Nonetheless, immunotherapy only benefits a subset of patients with cancer, and new approaches are needed. More specifically, only 15% of patients with metastatic head and neck squamous cell carcinoma (HNSCC) treated with anti–PD-1 therapy have shown a response (4), and very few responses were observed in patients with microsatellite stable colorectal cancer (CRC) (1). To increase the efficacy of immunotherapy in those patients, the cellular mechanisms leading to a successful antitumor immune response need to be identified. So far, the majority of studies have focused on the biology of CD8^+^ T cells, also known as killer T cells, and their role in the response against cancer. This focus is due in part to their capacity to recognize and kill tumor cells that present tumor antigen–derived peptides on their MHC class I molecules. Solid tumors display different levels of immune cell infiltration, and the presence of CD8^+^ T cells at the tumor site has been correlated with better overall survival (OS) and an improved response to checkpoint inhibitors in several cancer types (5–7). However, the CD8^+^ T cell infiltrate is heterogenous, and we and others have recently shown that only a fraction of the tumor-infiltrating lymphocyte (TIL) CD8^+^ T cells from HNSCC, melanoma, and CRC are specific for tumor antigens (8–11). More specifically, we found that tumor-reactive CD8^+^ T cells were enriched in cells that coexpressed the surface molecules CD39 and CD103 (double-positive [DP] CD8^+^) (8, 12). In addition, we found that the frequency of DP CD8^+^ T cells among total CD8^+^ TILs at the time of surgery correlated with better OS in a cohort of patients with HNSCC. Our work revealed that, while the presence of tumor-reactive CD8^+^ TILs can predict a positive outcome, other components of the immune response might be necessary to achieve a potent antitumor effect, leading to the eradication of the disease.

It is known from previous work that a successful immune response is the result of well-orchestrated cellular interactions between the different cell types involved (13). Hence, CD8^+^ T cells need to receive signals from other immune cells such as CD4^+^ Th cells to become fully licensed to kill tumor cells (14). Recent reports have provided evidence that CD4^+^ Th cells can recognize tumor-specific neoantigens in human solid tumors (15, 16), and their presence is associated with tumor regression in murine models (17) and in humans (18). In addition, Alspach et al. have shown in a sarcoma tumor model that CD4^+^ Th cells act together with CD8^+^ T cells to mount an efficient antitumor immune response (17). A greater understanding of the biology of tumor-reactive CD4^+^ Th cells would help define their role in the antitumor immune response. In addition, isolation of tumor antigen–specific CD4^+^ Th cells directly ex vivo, on the basis of cell-surface characteristics, would increase their potential for use in combined CD4^+^/CD8^+^ adoptive T cell therapy approaches. However, there is no consensus on which cell-surface markers allow their reliable isolation from a variety of different surgical specimens.

Here, we report that the coexpression of PD-1 and ICOS (DP CD4^+^) identified a unique population of CD4^+^ Th TILs found in the tumor microenvironment (TME). This subset of CD4^+^ Th TILs is present in HNSCC and CRC tumors at varying frequencies. The DP CD4^+^ TILs were highly activated in the tumor and had a distinct TCR repertoire. Finally, we found that DP CD4^+^ Th TILs were enriched for HPV-reactive and neoantigen-reactive T cells that were distinct from the epitopes recognized by the CD8^+^ T cells in the same patients. The limited overlap between the CD4^+^ and CD8^+^ antitumor responses suggest that they are complementary and might help overcome tumor heterogeneity. We believe our work will help design new therapeutic approaches to increase patients’ responses to immunotherapy treatments.

## Results

### PD-1 and ICOS expression by different subsets of CD4^+^ Th TILs in HNSCC and CRC tumors.

**Figure 1:**
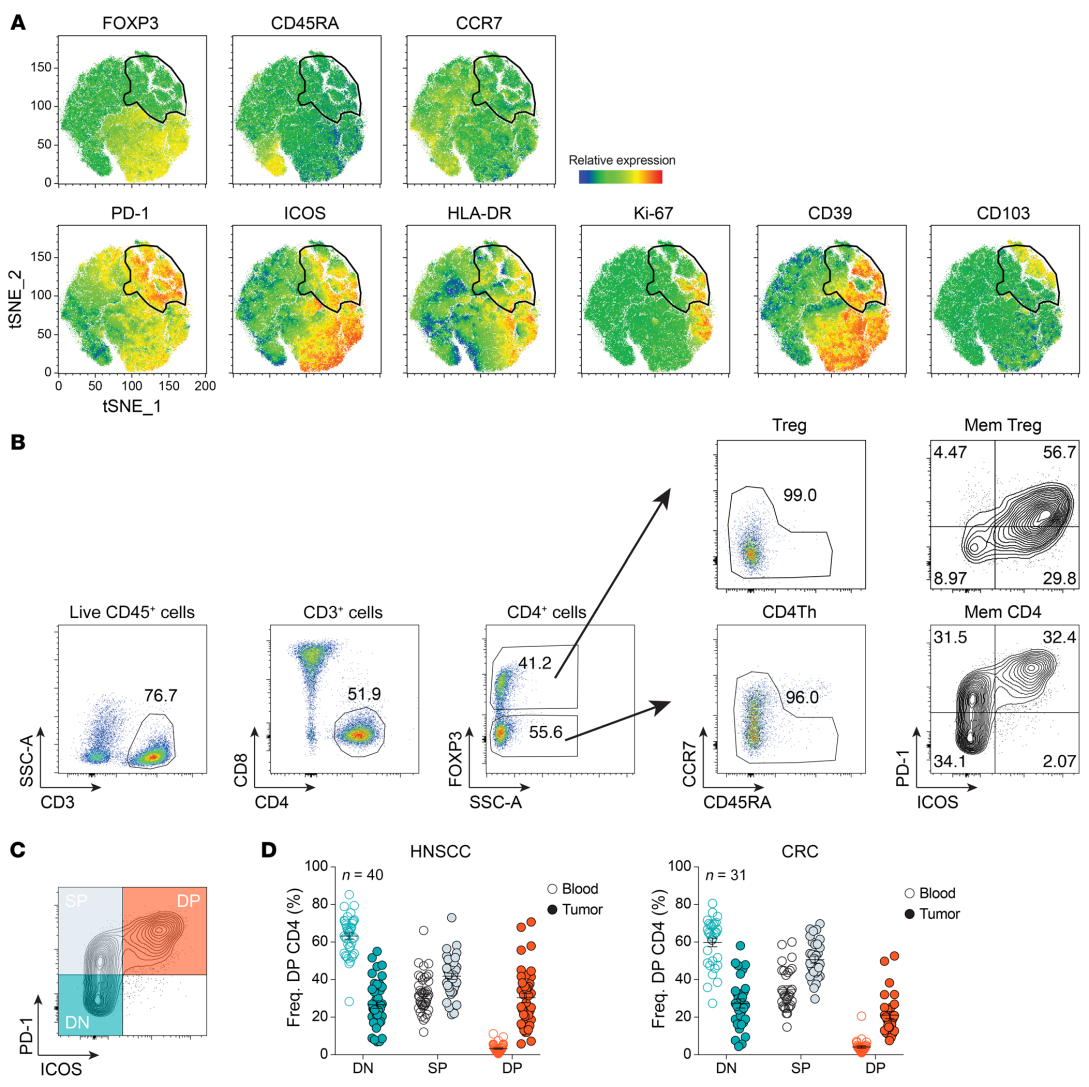
PD-1 and ICOS identify distinct subsets of CD4^+^ Th TILs. (**A**) t-SNE analysis of tumor-infiltrating CD3^+^CD4^+^ T cells isolated from 22 patients with HNSCC. The gate identified PD-1^+^ cells and gate was applied to all plots. (**B**) Flow cytometric analysis of CD4^+^ TILs and the gating strategy are shown for a representative patient with HNSCC. Mem, memory. (**C**) Subsets of CD4^+^ Th cells (DN, SP, and DP) were defined on the basis of differential expression of PD-1 and ICOS. (**D**) A summary of the frequency (Freq.) of DN, SP, and DP CD4^+^ Th cells in the blood and tumor of patients with HNSCC (left, *n =* 40) or CRC (right, *n =* 31) is shown. The DN (teal), SP (gray), and DP (orange) CD4^+^ Th cell subsets for each patient are highlighted on the graphs in **C** and **D**. Small horizontal lines indicate the mean ± SEM.

Our group and others have recently shown that CD8^+^ T cells isolated from human solid tumors comprise a heterogenous cell population, among which only a fraction of cells were tumor reactive (8, 9). We hypothesized that, similar to what has been shown for CD8^+^ T cells, CD4^+^ Th TILs contain a substantial proportion of bystander T cells. Therefore, we sought to identify cell-surface markers that enrich for tumor-reactive CD4^+^ Th cells. To this end, we used a high-dimensional flow cytometric assay to assess the heterogeneity and composition of the CD4^+^ TILs from 22 patients with HNSCC and 16 patients with CRC. Following t-distributed stochastic neighbor embedding (t-SNE) analysis, one of the most abundant clusters comprised cells expressing the transcription factor FOXP3, which is expressed by Tregs (Figure 1A and Supplemental Figure 1; supplemental material available online with this article; https://doi.org/10.1172/JCI156821DS1). This is in line with previous results showing that Tregs account for a large portion of the CD4^+^ TILs in HNSCC tumors (19). Among the FOXP3^–^ cells, we identified a population of CD4^+^ Th cells expressing high levels of PD-1. A proportion of these cells also expressed high levels of ICOS, HLA-DR, and CD39. Coexpression of PD-1 and ICOS had previously been shown to mark neoantigen-reactive CD4^+^ Th cells in a murine sarcoma tumor model (17). Hence, we further analyzed the expression pattern of PD-1 and ICOS by CD4^+^ Th TILs. This analysis revealed 3 distinct CD4^+^ Th cell populations: PD-1^−^ICOS^−^ double-negative (DN CD4^+^), PD-1^+^ICOS^–^ single-positive (SP CD4^+^), and PD-1^+^ICOS^+^ DP CD4^+^ T cells (Figure 1B). Of note, FOXP3^+^ Tregs expressed lower levels of PD-1 than did CD4^+^ Th TILs. Interestingly, we detected ICOS expression almost exclusively on PD-1–expressing cells among CD4^+^ Th TILs (Figure 1B). We found that the percentage of DP CD4^+^ Th cells was highly variable in patients with HNSCC, with a frequency ranging from 3.59% to 77.1% and a mean of 30.62% (Figure 1C). DP CD4^+^ Th TILs were also detected in patients with CRC, with a frequency ranging from 7.53% to 52.6% and a mean of 21.73% (Figure 1D). In contrast, we detected very few DP CD4^+^ Th cells in the peripheral blood of patients with HNSCC or CRC (Figure 1, C and D). The CD4^+^ Th cells in peripheral blood were mostly DN and SP CD4^+^ T cells.

### Tumor-derived DP CD4^+^ Th exhibit signs of activation and display a tumor-resident phenotype.

**Figure 2:**
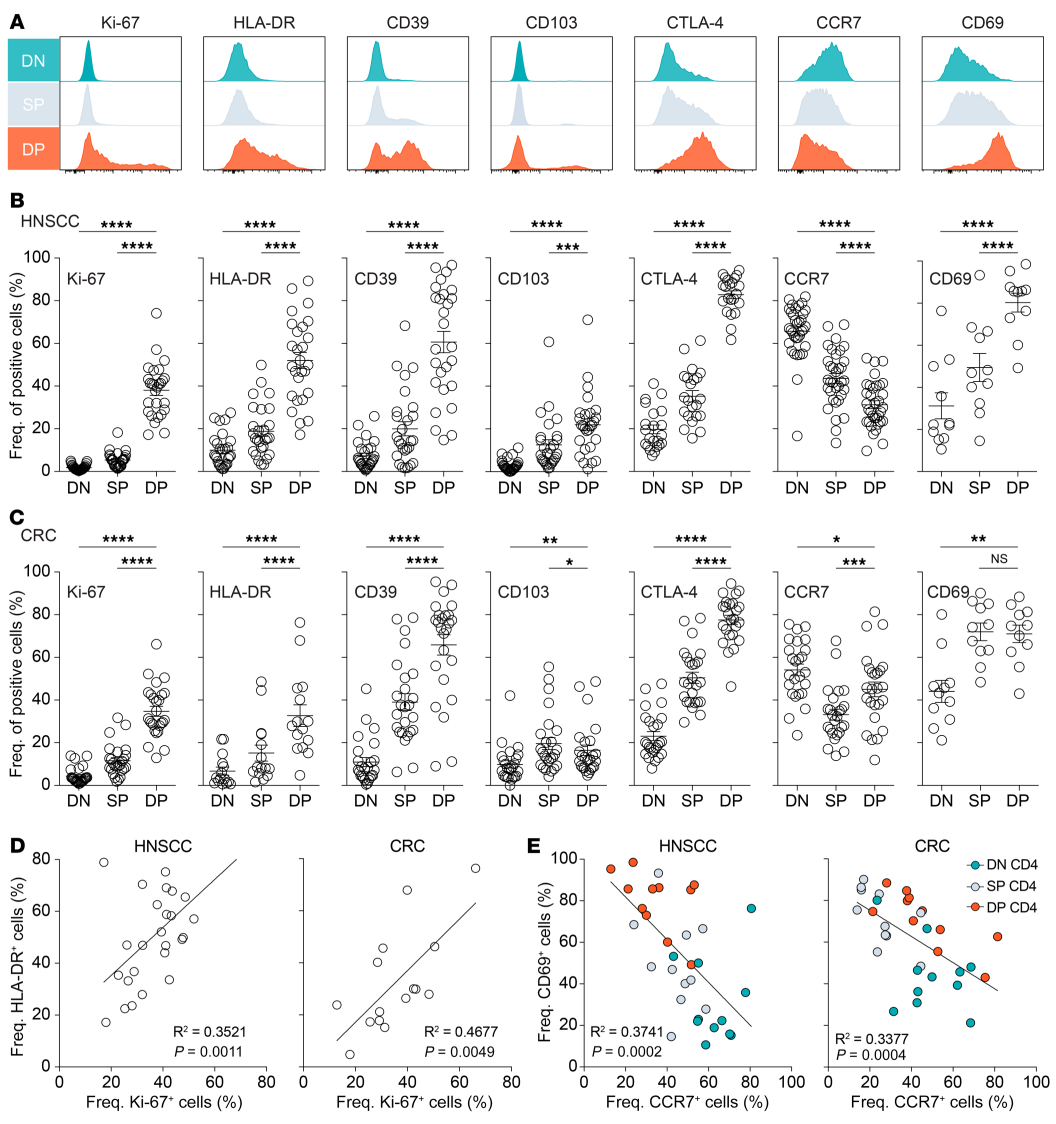
Phenotypic properties of DN, SP, and DP CD4^+^ Th TILs. (**A**) Ex vivo phenotypic analysis of the expression of Ki-67, HLA-DR, CD39, CD103, CTLA-4, CCR7, and CD69 on the 3 CD4^+^ TIL subsets of 1 representative patient with HNSCC. (**B**) Summary of the percentages of the above-mentioned markers among multiple patients with HNSCC (*n =* 27 for Ki-67, HLA-DR, CD39, and CD103; *n =* 22 for CTLA-4; *n =* 34 for CCR7; and *n =* 11 for CD69). (**C**) Summary of the percentages of the above-mentioned markers among multiple patients with CRC (*n =* 26 for Ki-67 and CCR7; *n =* 15 for HLA-DR; *n =* 25 for CD39, CD103, CTLA-4; and *n =* 11 for CD69). (**D**) Correlation between the percentages of HLA-DR^+^ cells and Ki-67^+^ cells in DP CD4^+^ TILs from patients with HNSCC (left, *n =* 27) or CRC (right, *n =* 15). (**E**) Correlation between the percentages of CD69^+^ cells and CCR7^+^ cells in the different CD4^+^ TIL subsets in patients with HNSCC (*n =* 11) or CRC (*n =* 11). Horizontal lines indicate the mean ± SEM. *P < 0.05, **P < 0.01, ***P < 0.001, and ****P < 0.0001, by 1-way ANOVA with Tukey’s post hoc test.

To better discern the activation and differentiation status of the DN, SP, and DP CD4^+^ Th TIL populations, we performed an in-depth phenotypic analysis using flow cytometry. The DP CD4^+^ Th TILs expressed higher levels of HLA-DR, CD39, and CTLA-4 compared with levels in the DN and SP CD4^+^ Th TILs (Figure 2A), in both HNSCC and CRC samples (Figure 2, B and C, respectively), indicative of an activated phenotype. CD39 expression was heterogenous in the DP CD4^+^ T cell population and was also expressed by a portion of SP CD4^+^ Th TILs, especially in the CRC samples. We observed a similar expression pattern for the integrin α E (CD103). The DP CD4^+^ Th TILs were proliferating in the tumor, as illustrated by increased frequencies of cells expressing Ki-67, which was mainly exclusive to that subset. The fraction of Ki-67^+^ cells correlated with the fraction of HLA-DR^+^ cells in the DP CD4^+^ T cell population in both tumor types (Figure 2D), however, the 2 markers were not always coexpressed. The DP CD4^+^ Th TILs also expressed high levels of CD69 and low levels of CCR7 (Figure 2E). CD69 is an activation molecule that is upregulated after T cell stimulation and antagonizes sphingosine 1 phosphate receptor 1–mediated (S1PR1-mediated) egress of T cells from tissue (20), whereas CCR7 is a chemokine receptor involved in T cell homing to draining lymph nodes (21–23). The expression pattern on DP CD4^+^ TILs suggests that these cells would remain in the TME. Conversely, DN CD4^+^ Th TILs expressed high levels of CCR7 and low levels of CD69, which would favor recirculation to other lymphoid tissues. Altogether, our phenotypic analysis showed that DP CD4^+^ Th TILs displayed a unique phenotype within the TME.

### DP CD4^+^ Th TILs contain cells from both the Th1 and Th17 lineage and display characteristics of follicular helper T cells.

**Figure 3:**
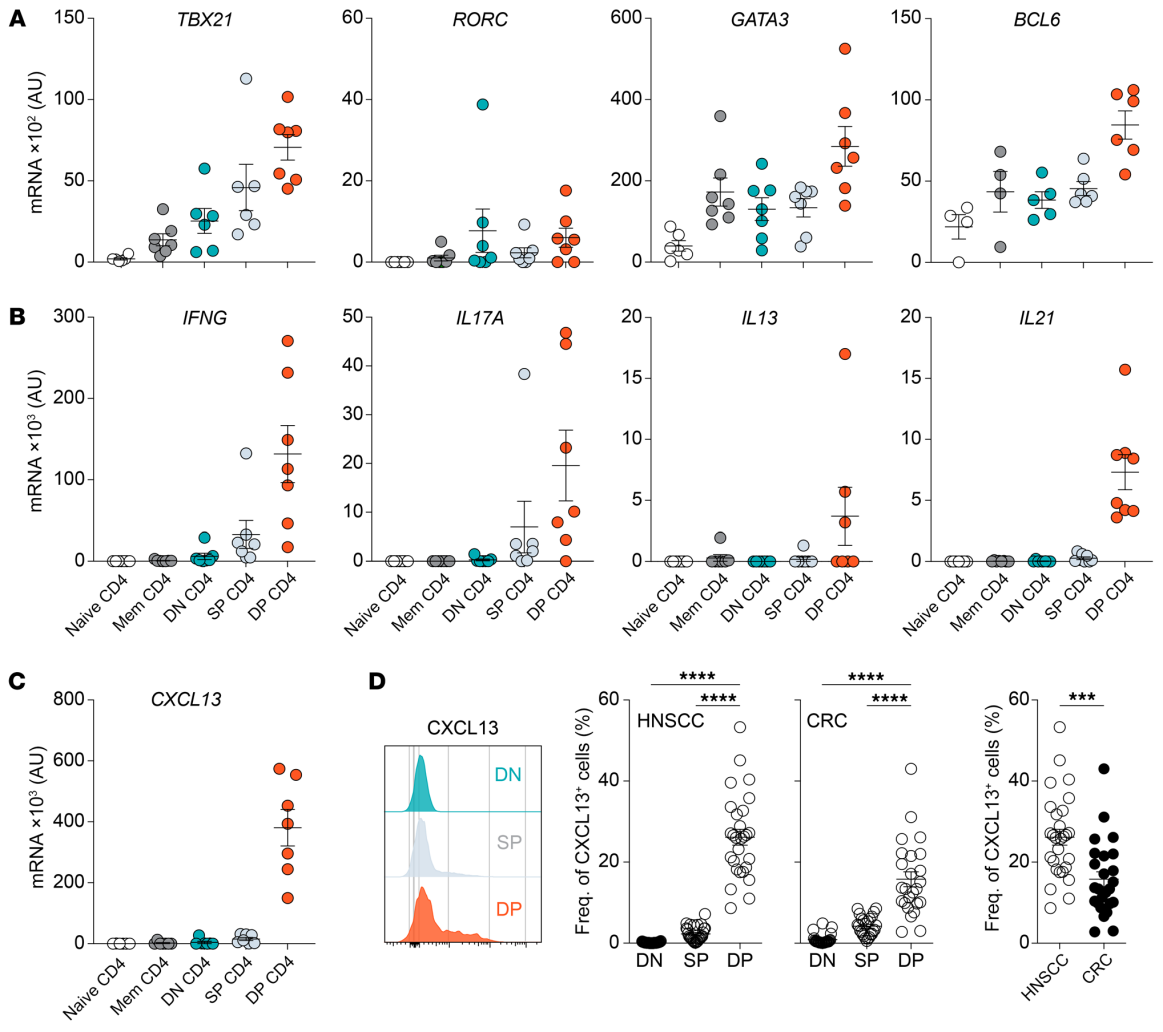
Functional properties of DN, SP, and DP CD4^+^ Th TILs. qPCR analysis of the expression of mRNA transcripts of transcription factors (**A**), cytokines (**B**), and CXCL13 (**C**) by the indicated CD4^+^ Th cell subsets isolated from patients with HNSCC or CRC directly ex vivo. (**D**) Left: Flow cytometric analysis of CXCL13 production by the different CD4^+^ TIL subsets directly ex vivo from 1 representative patient with HNSCC. Right: Summary of the frequency of CXCL13-producing cells in each CD4^+^ TIL subset in patients with HNSCC (*n =* 28) or CRC (*n =* 25). Horizontal lines indicate the mean ± SEM. ***P < 0.001 and ****P < 0.0001, by 1-way ANOVA with Tukey’s post hoc test (**D**, left and middle graphs) or unpaired *t* test (**D**, far right graph).

The differentiation and function of CD4^+^ Th cells are regulated by so-called “master” transcription factors that drive and maintain a unique transcriptional program, shaping the cell’s identity. Among those programs is the production of hallmark cytokines that influence cellular and humoral immune responses. To determine the identity of the DP CD4^+^ TILs in HNSCC and CRC, we measured transcripts of 3 master transcription factors directly ex vivo: T-bet, RORγt, and GATA-3 (Figure 3A). The DP CD4^+^ Th TILs expressed the Th1 transcription factor *TBX21* (T-bet) as well as the Th2 transcription factor *GATA3* (GATA-3), and mRNA transcripts for the Th17 transcription factor *RORC* (RORγt) were detected in 5 of the 7 patients analyzed. This expression pattern was mostly mirrored at the cytokine transcript level. The DP CD4^+^ Th TILs expressed mRNA transcripts for *IFNG*, *IL13*, and *IL17A*, with some interpatient variations (Figure 3B). Because PD-1 and ICOS are expressed by CD4^+^ Th cells involved in humoral responses, also known as follicular helper CD4^+^ T cells (Tfh cells), we measured transcripts for the Tfh-specific transcription factor Bcl6. We found that DP CD4^+^ Th TILs expressed the highest levels of *BCL6* compared with DN and SP cells and memory CD4^+^ Th cells in the blood. They also expressed mRNA transcripts for *IL21*, the signature cytokine produced by Tfh cells (Figure 3, A and B, and ref. 24). Similar to DP CD8^+^ TILs (Supplemental Figure 2, A and B) and PD-1^hi^CD8^+^ TILs (10), the DP CD4^+^ Th TILs also expressed high levels of *CXCL13* transcripts (Figure 3C), which was confirmed at the protein level in the absence of exogenous stimulation (Figure 3D). In HNSCC tumors, the mean frequency of DP CD4^+^ Th cells that expressed CXCL13 was 26.18% compared with only 15.78% in CRC tumors. Interestingly, we noted a positive correlation between the frequency of CXCL13-producing cells in the DP CD4^+^ and DP CD8^+^ TILs in both HNSCC and CRC tumors (Supplemental Figure 2, A and B). Collectively, DP CD4^+^ Th TILs are composed of cells from different Th cell lineages and display characteristics of Tfh cells.

### DP CD4^+^ Th TILs are found in proximity to MHC class II^+^ cells in the tumor stroma.

**Figure 4:**
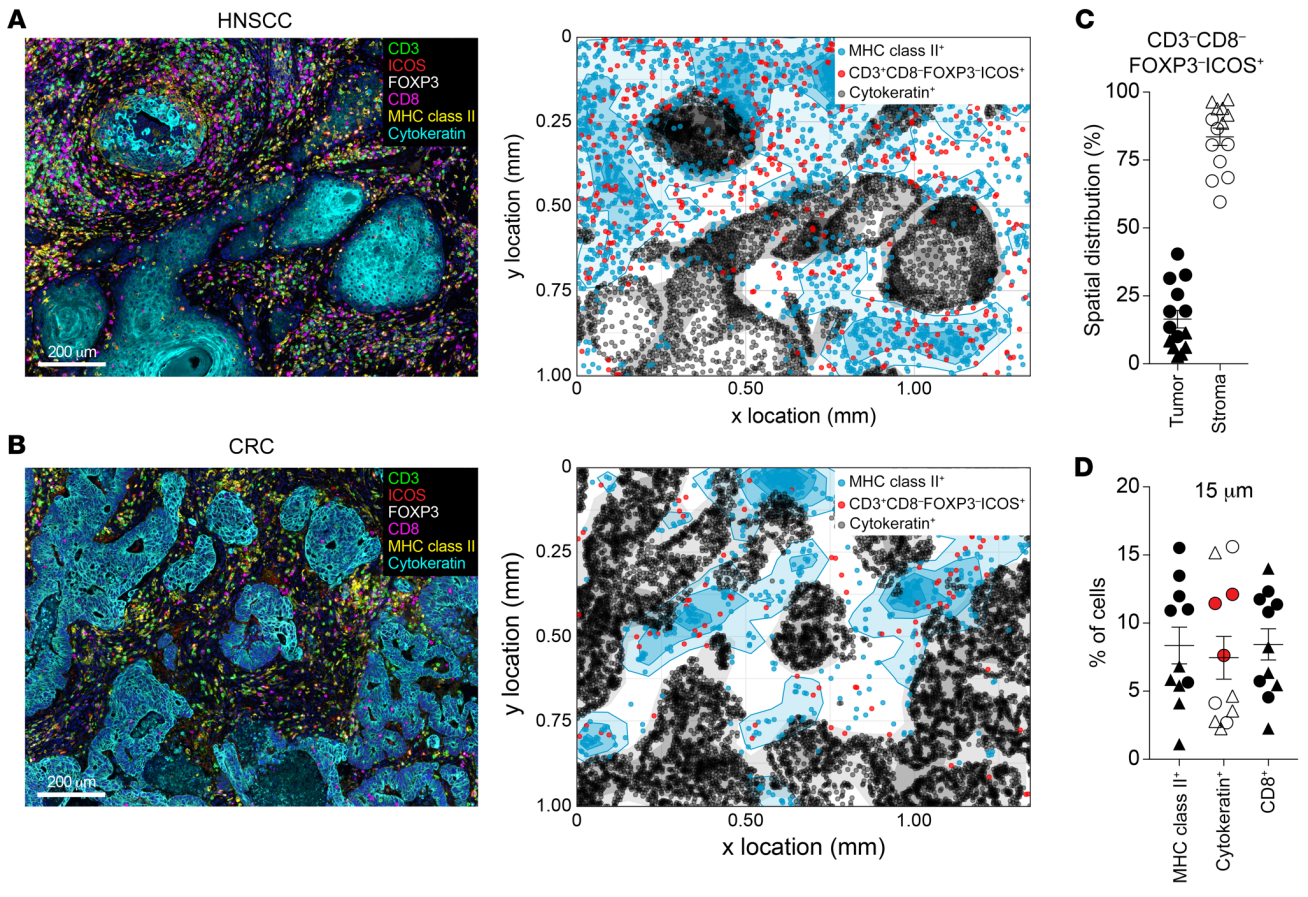
DP CD4^+^ Th cells are in the tumor stroma proximal to MHC class II^+^ cells. mIHC for CD3, CD8, FOXP3, ICOS, MHC class II, and cytokeratin on FFPE tissue sections from HNSCC and CRC tumors. Representative ROIs (left) from an HNSCC tumor (**A**) and a CRC tumor (**B**) and the corresponding cellular spatial relationship maps (right). Scale bars: 200 μm. The maps show cytokeratin^+^ cells (tumor) in black, MHC class II^+^ cells in blue, and CD3^+^CD8^–^FOXP3^–^ICOS^+^ (DP CD4^+^ Th cells) in red. (**C**) Analysis of the distribution of CD3^+^CD8^–^FOXP3^–^ICOS^+^ (DP CD4^+^ Th cells) in the tumor stroma and tumor epithelium in 14 tumors (*n =* 8 HNSCC and *n =* 6 CRC). (**D**) Neighbor’s cell analysis was performed on 11 tumors (*n =* 6 HNSCC and *n =* 5 CRC). The proportion of MHC class II^+^ cells in the stroma and cytokeratin^+^ cells detected within a 15 μm radius from CD3^+^CD8^–^FOXP3^–^ICOS^+^ (DP CD4^+^ Th) cells is indicated as the frequency of total cells. Circles represent HNSCC tumors, and triangles represent CRC tumors. Horizontal lines indicate the mean ± SEM. Data for patients with MHC class II–expressing tumor cells are shown in red in **D**.

To understand the role of DP CD4^+^ Th TILs in the antitumor immune response, we analyzed their spatial location and cellular interactions in the TME. Using multiplex immunohistochemistry (mIHC), we stained tumor sections from 8 patients with HNSCC and 6 patients with CRC. Figure 4, A and B, illustrate the results from 2 representative patients, 1 with HNSCC and 1 with CRC. After tissue segmentation into tumor stroma and tumor epithelium, we determined the proportion of DP CD4^+^ Th cells, as defined by CD3^+^CD8^–^FOXP3^–^ICOS^+^ expression, in these 2 spatial areas. We observed that, even though some DP CD4^+^ Th cells could be detected in the tumor epithelium, 80% of the cells were present in the tumor stroma (Figure 4C). Because of their highly activated phenotype and proliferative status in the TME, we hypothesized that the DP CD4^+^ Th cells recognize their cognate antigen locally and thus should be present near MHC class II^+^ antigen-presenting cells (APCs). A neighbor’s cell analysis in tumor samples from 11 patients (*n* = 6 samples from patients with HNSCC; *n* = 5 samples from patients with CRC; Figure 4D) indicated that a portion of the DP CD4^+^ Th cells were detected close to MHC class II^+^ cells in the TME. Of note, there was a trend toward a higher proportion of DP CD4^+^ cells in proximity to MHC class II^+^ cells in HNSCC tumors (circles) as compared with that seen in CRC tumors (triangles). In addition, for 3 patients with MHC class II–expressing tumor cells (highlighted in red on the graph), we detected an increased frequency of DP CD4^+^ Th cells infiltrating or present near the tumor epithelium. Besides MHC class II^+^ cells, CD8^+^ T cells were also present in the vicinity of the DP CD4^+^ cells, which suggests that the latter could influence the biology of CD8^+^ TILs.

### A positive correlation between DP CD4^+^ and DP CD8^+^ TIL subsets in HNSCC, but not CRC.

**Figure 5:**
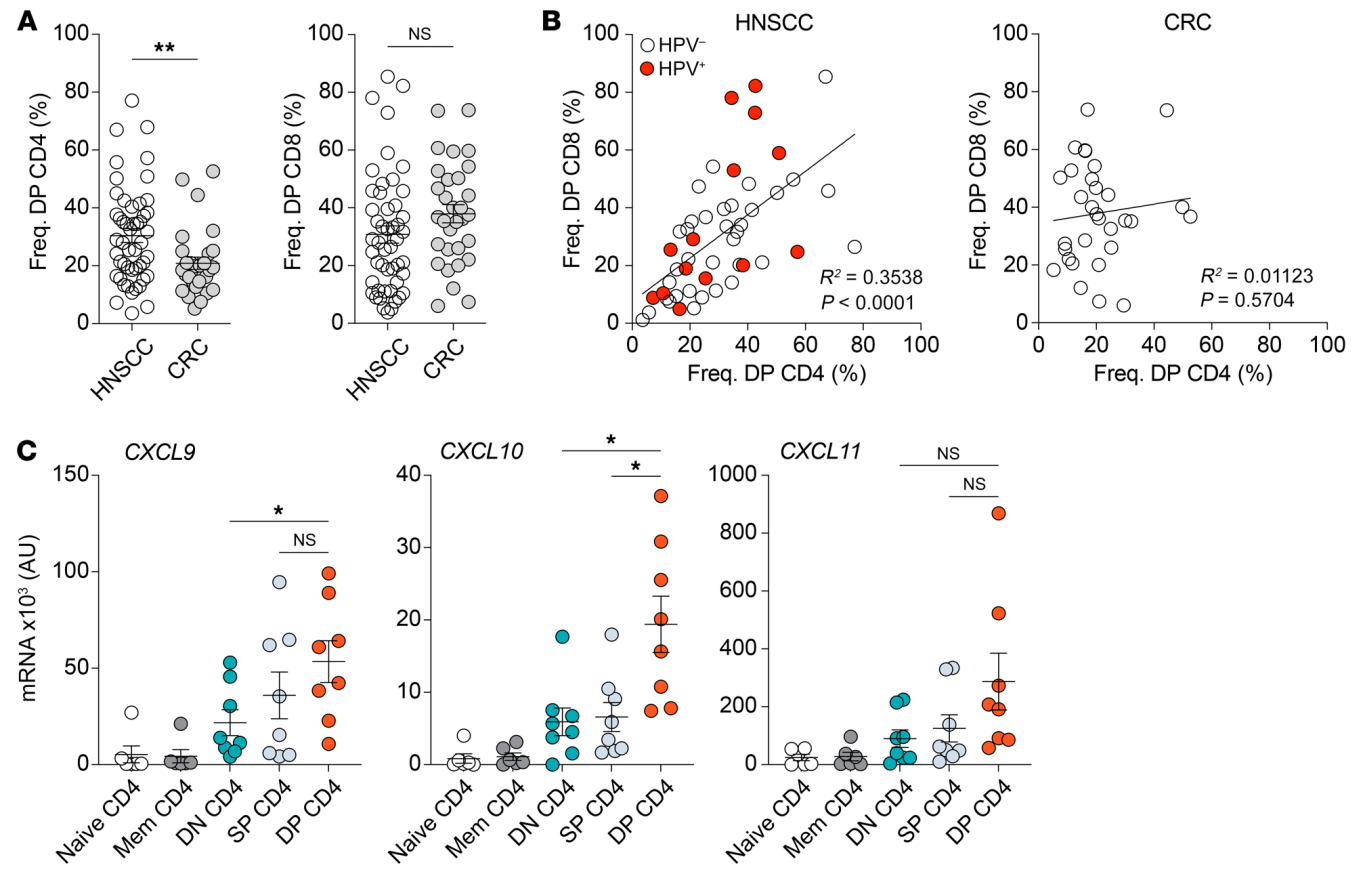
The presence of DP CD4^+^ Th cells positively correlates with DP CD8^+^ T cells in HNSCC, but not in CRC. (**A**) Comparison of the percentage of DP CD4^+^ and DP CD8^+^ cells between HNSCC (*n =* 49) and CRC (*n =* 31) tumors. (**B**) Correlation between the frequencies of DP CD4^+^ and DP CD8^+^ cells in patients with HNSCC (*n =* 49) or CRC (*n =* 31). Red circles represent HPV^+^ HNSCC tumors. (**C**) qPCR analysis of *CXCL9*, *CXCL10*, and *CXCL11* mRNA expression by the indicated CD4^+^ T cell subsets isolated from patients with HNSCC or CRC directly ex vivo. Horizontal lines indicate the mean ± SEM. *P < 0.05 and **P < 0.01, by unpaired 2-tailed *t* test (**A**) or 1-way ANOVA with Tukey’s post hoc test (**C**).

Based on our data and the study from Alspach et al. (17), we next sought to determine whether the DP CD4^+^ Th TILs influenced the biology of CD8^+^ TILs, in particular CD39^+^CD103^+^ (DP) CD8^+^ T cells, which are highly enriched for tumor-reactive cells (8, 11, 12). First, we compared the frequency of DP CD4^+^ and DP CD8^+^ TILs in HNSCC and CRC tumor samples. We found that the median frequency of DP CD4^+^ Th TILs was significantly higher in HNSCC tumors, whereas there was no difference for the DP CD8^+^ TILs between the 2 tumor types (Figure 5A). Next, we did a side-by-side comparison of the frequency of DP CD4^+^ Th TILs and DP CD8^+^ TILs in HNSCC and CRC tumors, respectively. The frequency of DP CD4^+^ Th TILs positively correlated with the proportion of DP CD8^+^ TILs in HNSCC tumors, independent of the HPV status of the tumors (Figure 5B). In contrast, we found no correlation in the CRC tumors. Besides cytokines, which can activate and regulate other cell types, CD4^+^ Th cells can also produce chemokines that participate in the recruitment of immune cells. Among the chemokines known to attract CD8^+^ T cells into the tumor are CXCL9, CXCL10, and CXCL11, all of which bind to the chemokine receptor CXCR3 expressed at the surface of CD8^+^ T cells. The level of RNA transcripts for these 3 chemokines, as measured directly ex vivo by quantitative PCR (qPCR), showed that the DP CD4^+^ Th TILs had significant upregulated expression of *CXCL9* and *CXCL10* as compared with the other CD4^+^ Th TIL populations (Figure 5C). Hence, our results suggest that DP CD4^+^ Th TILs might attract CXCR3^+^ cells within the TME.

### DP CD4^+^ Th cells have an oligoclonal TCR repertoire distinct from the other CD4^+^ Th TIL subsets.

**Figure 6:**
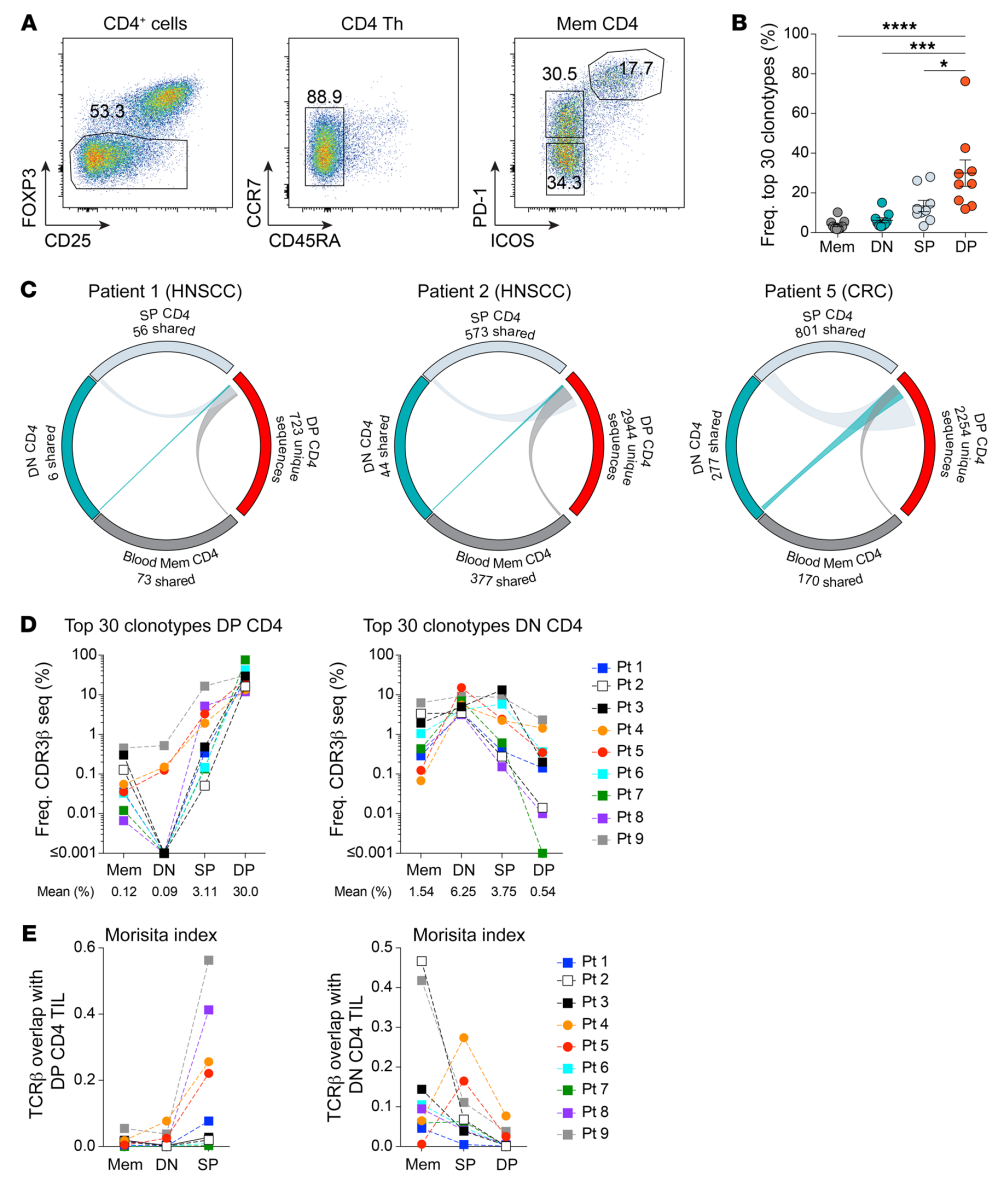
DP CD4^+^ Th TILs have a unique and oligoclonal TCR repertoire. (**A**) Gating strategy for the TCR repertoire analysis. (**B**) Cumulative frequencies of the top 30 clonotypes for each CD4^+^ Th subset in 9 patients with cancer. (**C**) Circos plots of unique productive TCRβ nucleotide sequences from each of the indicated cell subsets. Connections highlight sequences from DP CD4^+^ TILs found in the other CD4^+^ Th cell populations. The number of shared sequences is indicated. Circos plots for 2 patients with HNSCC and 1 patient with CRC are shown. (**D**) Cumulative frequencies of the top 30 clonotypes from DP CD4^+^ TILs (left) and DN CD4^+^ TILs (right) in 9 patients (Pt) with cancer. Their frequencies in blood memory CD4^+^, DN, SP, and DP CD4^+^ TILs are represented. (**E**) Similarity between the TCR repertoires of the different CD4^+^ T cell subsets was measured using the Morisita-Horn index for 9 patients with cancer. Each color represents a distinct patient connected with a dashed line (**D** and **E**). Horizontal lines indicate the mean ± SEM. *P < 0.05, ***P < 0.001, and ****P < 0.0001, by 1-way ANOVA with Tukey’s post hoc test (**B**).

Thus far, the data show that the DP CD4^+^ Th TILs are highly activated and proliferating in the TME, suggesting recognition of their cognate antigen in the tumor. If this assumption is correct, we should observe selective expansion of dominant T cell receptor (TCR) clonotypes within the DP CD4^+^ Th TILs. To explore this hypothesis, we sorted memory CD4^+^ T cells from the blood (blood memory CD4) as well as DN, SP, and DP CD4^+^ Th TILs, while gating on FOPX3^–^ cells to exclude Tregs (Figure 6A). Sequence analyses of the CDR3 region in the *TRB* genes revealed differences in the composition of the TCR repertoire between the DP CD4^+^ Th TILs and blood memory CD4^+^, DN, and SP CD4^+^ Th TILs (Figure 6B). The top 30 clonotypes accounted for 30% of the DP CD4^+^ TIL repertoire (range: 13.42–76.25), whereas the top 30 clonotypes in the other CD4^+^ Th TIL subsets only represented 3.92% (range: 1.83–10.23), 6.25% (range: 3.46–15.08), and 13.47% (range: 3.31–28.07) of the blood memory CD4^+^, DN, and SP CD4^+^ Th TIL repertoires, respectively. Next, we analyzed the TCR repertoire overlap of the DP CD4^+^ Th TILs with the other CD4^+^ T cell subsets (Figure 6C). A majority of the clonotypes within the DP CD4^+^ Th TILs were unique to this subset, and there was little overlap with the SP CD4^+^ Th TILs and even less with the DN CD4^+^ Th TILs and blood memory CD4^+^ T cells. In addition, the dominant clonotypes in the DP CD4^+^ Th TILs were present at much lower frequencies or not detected in the other CD4^+^ T cell subsets (Figure 6D). Calculation of the Morisita index, an abundance-based similarity index that determines the overlap between 2 cell populations, further supported these findings, showing consistent results across 9 patients (Figure 6E). In contrast to the DP CD4^+^ Th TILs, the TCR repertoire of DN CD4^+^ Th TILs preferentially overlapped with the blood memory CD4^+^ T cells. These data suggest that the DN CD4^+^ Th TILs represent a population of CD4^+^ T cells that recirculate between the tumor and the periphery (Figure 2E). Collectively, our results support the idea of an antigen-driven stimulation of CD4^+^ Th TILs at the tumor site that leads to local activation and expansion of DP CD4^+^ cells.

### DP CD4^+^ Th TILs contain T cells that recognize tumor-associated antigens and tumor-specific neoantigens.

**Figure 7:**
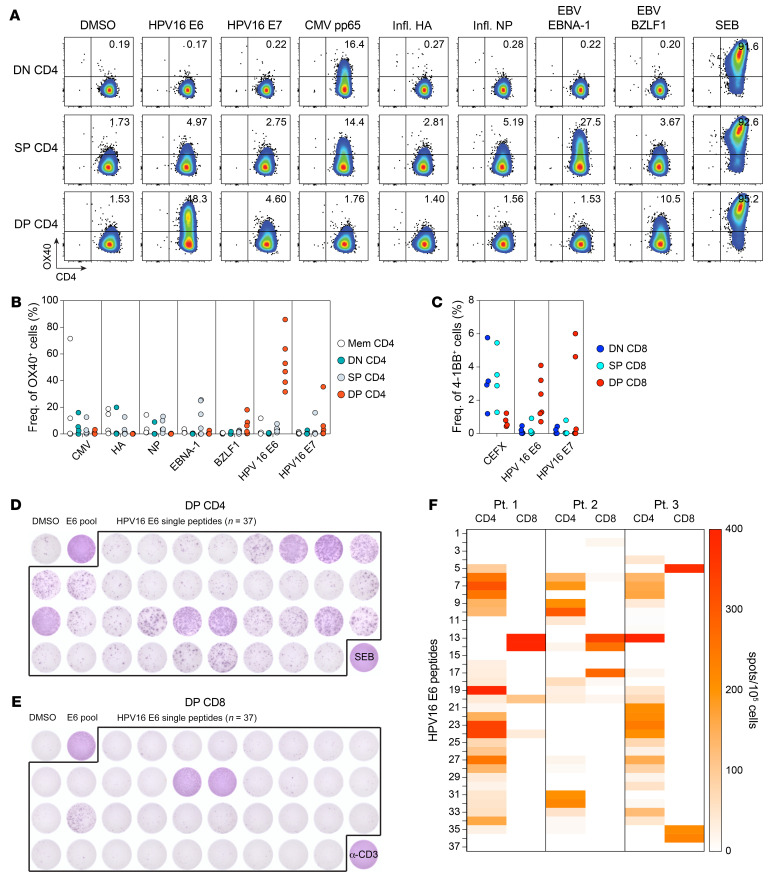
DP CD4^+^ Th TILs recognize HPV-associated antigens. (**A**) In vitro–expanded CD4^+^ TIL subsets (DN, SP, and DP) were cocultured with autologous B cells pulsed with DMSO or the indicated peptide pools. T cell reactivity was assessed by OX40 upregulation after 24 hours of culturing. Data for 1 representative patient with HPV^+^ HNSCC are shown. (**B**) Summary of the reactivity of CD4^+^ Th subsets to the indicated peptide pools for 6 patients with HPV^+^ HNSCC. (**C**) Summary of the reactivity of CD8^+^ T cells to HPV16 E6, HPV16 E7, and CEFX peptide pools for the same 6 patients with HPV^+^ HNSCC. CD8^+^ T cell activation was assessed by 4-1BB upregulation. DP CD4^+^ (**D**) and DP CD8^+^ (**E**) TILs isolated from 1 patient with HPV^+^ HNSCC were cultured with autologous B cells pulsed with DMSO, the HPV16 E6 peptide pool, or the individual overlapping peptides contained in the HPV16 E6 peptide pool (*n =* 37). Reactivity was measured by IFN-γ ELISPOT assay. SEB or anti-CD3 antibodies were used as positive controls for CD4^+^ and CD8^+^ T cells, respectively. (**F**) Summary of the reactivity of DP CD4^+^ and DP CD8^+^ TILs to HPV16 E6 individual peptides for 3 different patients with HPV^+^ HNSCC, as measured by IFN-γ ELISPOT assay. The colors in the heatmap legend represent the number of detected spots/10^5^ cells.

Both the proliferative state and clonal expansion, as well as their presence near MHC class II^+^ cells in the TME suggest that DP CD4^+^ Th TILs recognize tumor antigens. To address this hypothesis, we pursued 2 different approaches. First, we analyzed the T cell response to HPV proteins in patients with HPV-associated HNSCC, more specifically the response against the viral proteins E6 and E7, which are directly associated with tumor development (25) and are conserved between patients. CD4^+^ Th TIL subsets were sorted on the basis of differential expression of PD-1 and ICOS and then expanded. We screened the DN, SP, and DP CD4^+^ subsets for reactivity to HPV16 E6 and E7 peptide pools and measured the upregulation of the activation marker OX40 as well as IFN-γ secretion by ELISPOT. In all 6 patients analyzed, we detected reactivity to HPV antigens (Figure 7, A and B). Interestingly, E6- and/or E7-reactive T cells were strongly enriched in the DP CD4 Th TILs. Of note, responses directed against the E6 peptide pool were predominant, with only 1 patient showing a CD4 response to the E7 peptide pool. In contrast, T cells specific for nontumor-related viruses such as CMV, flu, and EBV were detected at higher frequencies in the DN and SP CD4^+^ cell subsets. CD4^+^ Th cells play crucial roles in the development of effector cells and are essential for maintaining memory CD8^+^ T cells (14). Thus, we also investigated the presence of HPV-reactive T cells in the CD8 compartment in the same patients. Screening of expanded CD8^+^ TILs sorted on the basis of CD39 and CD103 expression with peptide pools or in vitro–transcribed RNA for E6 and E7 demonstrated strong enrichment of HPV-specific T cells in the DP CD8^+^ TILs, as revealed by 4-1BB upregulation and IFN-γ secretion (Figure 7C). Instead, CD8^+^ T cells recognizing CMV, EBV, or flu peptides were detected primarily in the DN and SP CD8^+^ TILs. Interestingly, the frequency of HPV-reactive T cells in the DP CD8^+^ cells was on average 5- to 10-fold lower than the frequency of HPV-reactive DP CD4^+^ cells. Next, we compared the breadth of the response against HPV16 E6 in DP CD4^+^ and DP CD8^+^ TILs (Figure 7, D and E). Assessment of the reactivity of DP CD4^+^ TILs to single peptides showed that the CD4 response against HPV16 E6 was specific for multiple epitopes within the E6 protein. However, the majority of the CD8^+^ T cell response was specific for only 1 or 2 dominant epitopes, resulting in an antigenic response with restricted breadth. The epitopes recognized by DP CD8^+^ TILs were distinct from those recognized by DP CD4^+^ TILs in all 3 patients analyzed (Figure 7F).

**Figure 8:**
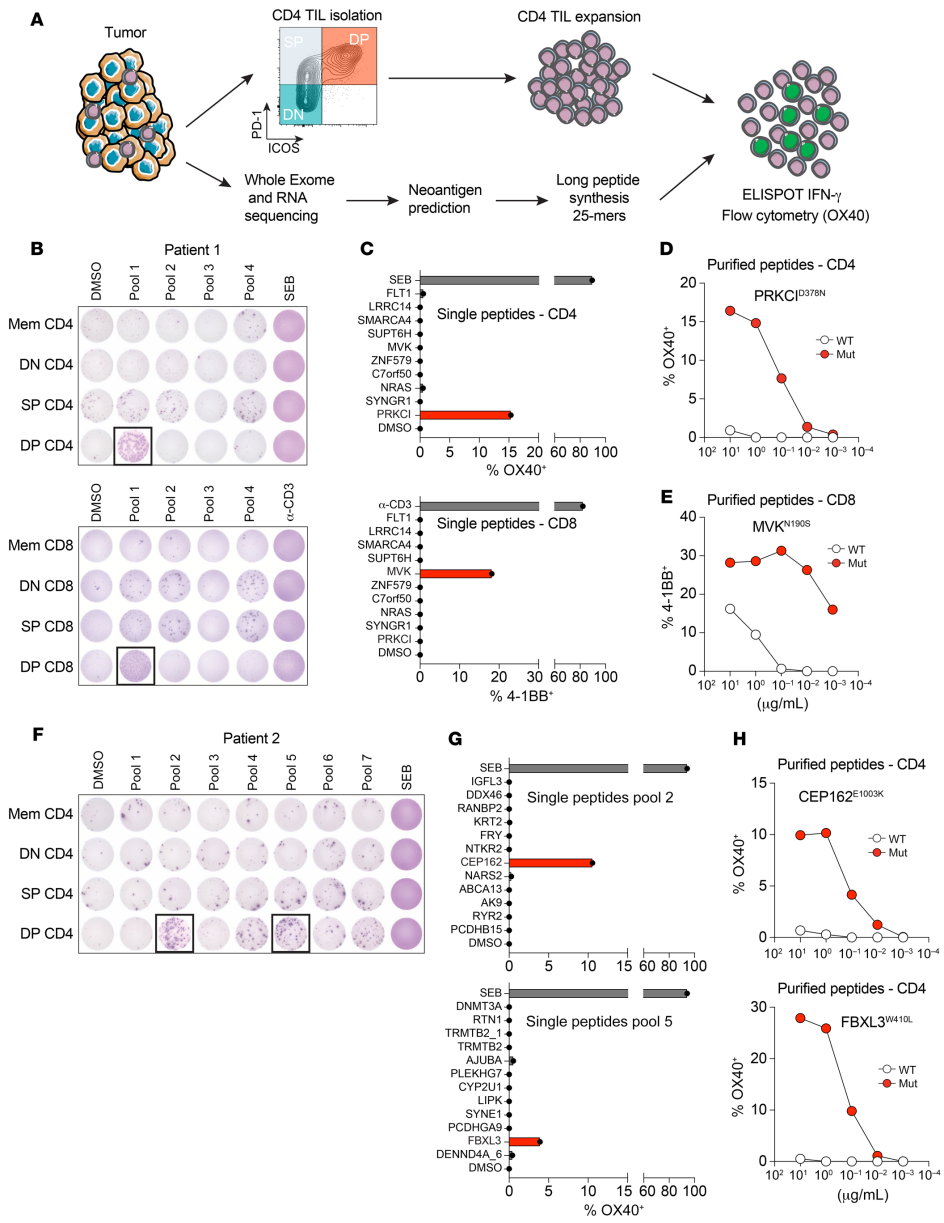
DP CD4^+^ Th TILs recognize tumor-specific neoantigens. (**A**) Schematic representation of the methodology used to identify neoantigen-reactive CD4^+^ Th cells. (**B**) In vitro–expanded CD4^+^ and CD8^+^ T cell subsets (DN, SP, and DP) from patient 1 were cocultured with autologous B cells pulsed with DMSO or the indicated peptide pools. T cell reactivity was measured by IFN-γ ELISPOT assay. (**C**) Reactivity of DP CD4^+^ and DP CD8^+^ TILs to B cells pulsed with individual 25 mer peptides from pool 1. Flow cytometric analysis of OX40 or 4-1BB upregulation on CD4^+^ or CD8^+^ T cells, respectively is shown. The mutations recognized are highlighted in red. (**D**) DP CD4^+^ Th TILs were cocultured with autologous B cells pulsed with decreasing concentrations of WT or mutated (Mut) PRKCI^D378N^ 25 mer peptides. Reactivity was measured by flow cytometric analysis of OX40 upregulation on CD4 cells. (**E**) DP CD8^+^ TILs were cocultured with autologous B cells pulsed with decreasing concentrations of WT or mutated 8 mer MVK^N190S^ peptides. Reactivity was measured by flow cytometric analysis of 4-1BB upregulation on CD8^+^ T cells. (**F**) In vitro–expanded CD4^+^ T cell subsets (DN, SP, and DP) from patient 2 were cocultured with autologous B cells pulsed with DMSO or the indicated peptide pools. T cell reactivity was measured by IFN-γ ELISPOT assay. (**G**) Reactivity of DP CD4^+^ Th TILs to B cells pulsed with individual 25 mer peptides from peptide pools 2 and 5. Flow cytometric analysis of OX40 upregulation on CD4^+^ cells is shown. The mutations recognized are highlighted in red. (**H**) DP CD4^+^ Th TILs were cocultured with autologous B cells pulsed with decreasing concentrations of WT or mutated CEP162^E1003K^ or FBXL3^W410L^ 25 mer peptides. Reactivity was measured by flow cytometric analysis of OX40 upregulation on CD4^+^ cells.

In a second, complementary approach, we analyzed the response to neoantigens derived from nonsynonymous tumor somatic mutations in 4 patients with cancer (Figure 8A and Supplemental Figure 3). We performed whole-exome sequencing (WES) for 2 patients with HPV^+^ HNSCC, 1 patient with HPV^–^ HNSCC, and 1 patient with microsatellite stable (MSS) CRC. All patients had a low-to-moderate tumor mutational burden (between 1.11 and 2.98 mut/megabase) (Supplemental Table 1). For patient 1, who had an HPV^+^ HNSCC tumor, we predicted and screened 28 variants (covered by 37 peptides) in 4 different pools. Tumor-reactive IFN-γ–secreting T cells were detected in pool 1, and the reactive cells were enriched in the DP CD4^+^ Th TIL subset (Figure 8B). Testing of the individual peptides from that pool revealed reactivity to a single mutation in the PRKCI gene (Figure 8C). This reactivity was confirmed by comparing a titration of both HPLC-purified WT and mutated peptides (Figure 8D). Following a similar approach, we screened 74 variants (covered by 82 peptides) for patient 2, who had HPV^–^ HNSCC, in 7 different pools. The main responses were identified in pool 2 and pool 5, predominantly in the DP CD4^+^ Th TILs (Figure 8F). After deconvoluting the pools, we found reactivity to 2 unique mutations, 1 in the CEP162 gene and 1 in the FBXL3 gene (Figure 8G). Both reactivities were confirmed to be neoantigen specific by comparing the mutated peptides and WT peptides (Figure 8H). For the third patient (patient 3), who had HPV^+^ HNSCC, we screened 49 variants (covered by 53 peptides) in 5 pools. T cell reactivity was restricted to pool 3 and only detected in the DP CD4^+^ Th TILs (Supplemental Figure 3A). The single mutation was present in the TTC17 gene, and the DP CD4^+^ Th TILs only recognized the mutated, but not the WT, peptide (Supplemental Figure 3, B and C). Finally, we analyzed tumor reactivity in 1 patient with CRC for whom we screened 57 variants (covered by 68 peptides) in 7 pools. Similar to the 3 patients with HNSCC, we observed reactivity in pool 3 primarily in the DP CD4^+^ Th TILs (Supplemental Figure 3F). The DP CD4 Th TILs recognized a single somatic mutation in the PRTM1 gene, which was confirmed to be neoantigen specific (Supplemental Figure 3G). In parallel, we analyzed the reactivity of DP CD8^+^ TILs against tumor-specific somatic mutations in patients 1 and 3. For patient 1, IFN-γ secretion and flow cytometric upregulation of 4-1BB demonstrated reactivity in pool 1 uniquely in the DP CD8^+^ cells (Figure 8B). To identify the specific neoantigen being recognized, DP CD8^+^ TILs were cultured with the 10 individual peptides contained in pool 1. Our analysis indicated that DP CD8^+^ TILs recognized a unique mutation in the MVK gene (Figure 8C). This reactivity was neoantigen specific, as 4-1BB upregulation was still detectable with very low concentrations of the mutated peptide (Figure 8E). For patient 3, we used the tandem minigene (TMG) approach for the first screening. After coculturing with APCs electroporated with TMGs, we detected reactivity in TMG 1 and TMG 2 only in the DP CD8^+^ cells (Supplemental Figure 3D). The analysis of individual peptides revealed that DP CD8^+^ cells recognized 2 unique single mutations, 1 in TMG1 (YAP1) and 1 in TMG2 (TRAM1) (Supplemental Figure 3E). The specific reactivity against YAP1 was confirmed by comparing the response to the WT and mutated peptides, with IFN-γ secretion observed only for the latter (Supplemental Figure 3F). In contrast, both the WT and mutated peptides for TRAM1 were recognized by DP CD8^+^ T cells, suggesting potential self-reactivity. Altogether, for the 4 patients analyzed, we were able to identify at least 1 population of neoantigen-reactive CD4^+^ T cells in each patient, and those cells were present predominantly in the DP CD4^+^ Th TILs.

## Discussion

In this study, we show that among CD4^+^ TILs, the cells that coexpressed PD-1 and ICOS were enriched for tumor-reactive T cells in patients with HNSCC or CRC. The DP CD4^+^ Th cells were primarily found in the TME, had an activated phenotype, and were proliferating in the tumor. Furthermore, we show that the DP CD4^+^ Th TILs were enriched in T cells that recognize tumor-associated antigens such as HPV16 E6 and E7 proteins in HPV-related HNSCC as well as tumor-specific neoantigens arising from nonsynonymous somatic mutations. Finally, our results show that tumor-reactive CD4^+^ and CD8^+^ T cells recognized distinct epitopes, suggesting that CD4^+^ and CD8^+^ T cells might play a complementary role in the antitumor response. Identifying ways to harness the cellular cooperation between these 2 CD4^+^ and CD8^+^ TIL subsets could lead to more efficient tumor recognition and control and could potentially aid in tumor destruction.

A recent study showed that CD4^+^ TILs recognizing HPV-related epitopes were enriched for cells expressing CD39 (26). While, on average, 60% of CD39^+^ CD4^+^ Th cells have the DP CD4^+^ phenotype, our results demonstrated that the remaining cells (DN CD4^+^ and SP CD4^+^) were not enriched in tumor-reactive TCRs. In addition, approximately two-thirds of the CD39^+^CD4^+^ T cells in the tumor were Tregs, which is important information to consider before using this marker to isolate and expand cells for adoptive T cell therapy. Thus, our work further refines the phenotype of tumor-reactive CD4^+^ Th cells in human solid tumors. Our proposed strategy to enrich for human tumor–reactive CD4^+^ Th cells is supported by a previous study in a mouse sarcoma model showing that neoantigen-reactive CD4^+^ Th cells found in the tumor expressed both PD-1 and ICOS (17).

We and others have shown that the CD8^+^ T cell compartment in the TME is heterogenous and that only a fraction of the CD8^+^ TILs recognized tumor antigens (8–10, 27). Similarly, our current study indicates that tumor-reactive CD4^+^ TILs were enriched in the DP CD4^+^ subset. The TCR specificities of the other infiltrating CD4^+^ Th cells (DN and SP) were directed against nontumor-related antigens such as common viral antigens from CMV, EBV, and flu. Therefore, using PD-1 and ICOS to enrich for tumor-reactive CD4^+^ Th cells prior to TIL expansion may be a way to increase the clinical success of adoptive T cell therapy. This strategy will allow for the identification of TCR sequences within the DP CD4^+^ Th TILs that recognize mutated or shared tumor antigens bound to MHC class II molecules that could then be used in TCR engineering for personalized therapies in patients with cancer. Considering the “helper” role of the CD4^+^ Th cells in the immune response, this strategy might also aid in the evaluation of the cotransfer of antigen-specific CD4^+^ and CD8^+^ T cells to improve adoptive T cell therapy approaches.

In contrast to CD8^+^ T cells, CD4^+^ Th cells recognize their cognate antigen presented by MHC class II molecules. MHC class II expression is found predominantly on myeloid cells, B cells, DCs, and activated CD4^+^ and CD8^+^ T cells; however, in some cases tumor cells express MHC class II, which raises the question of whether DP CD4^+^ Th TILs can recognize their cognate antigen directly in the TME. Our spatial analysis revealed that DP CD4^+^ Th TILs could be found in the area of the TME with a high density of MHC class II^+^ cells. Moreover, DP CD4^+^ Th TILs displayed an activated phenotype and were proliferating in situ, as illustrated by HLA-DR and Ki-67 expression, respectively. Taken together, this suggests that DP CD4^+^ Th TILs can recognize their cognate antigen in the TME. To dissect the role of CD4^+^ Th cells in antitumor immunity, it will be important to define the nature of the MHC class II^+^ cells interacting with the DP CD4^+^ cells in the TME and determine the influence of those interactions on the antitumor immune response. It will also be important to identify cytokines/chemokines secreted by the DP CD4^+^ Th cells following TCR stimulation and their impact on surrounding immune cells in the TME. Identifying key cellular interaction networks and the relevant soluble factors might lead to the development of new drug targets that could boost the function of the DP CD4^+^ cells and help recruit these cells from the stroma into the tumor area.

PD-1^+^ and ICOS^+^ TILs are enriched for tumor-reactive CD4^+^ Th cells. This phenotype is associated with CD4^+^ Tfh cells that are characterized by the secretion of CXCL13, a chemokine that binds to CXCR5 to recruit B cells and other CXCR5^+^ T cells (24). Tfh cells are usually present in the B cell follicles in secondary lymphoid organs, where they interact with B cells and are involved in the germinal center (GC) reaction. CXCL13-producing CD4^+^ Th cells have been reported in patients with breast cancer, in whom they were enriched in tertiary lymphoid structures, and their presence correlated with B cell infiltration and GC maturation at the tumor site and was associated with improved prognosis (28, 29). Here, we show that DP CD4^+^ Th cells isolated from HNSCC and CRC tumors expressed mRNA transcripts for the Tfh transcription factor Bcl6 and their signature cytokine IL-21 and could produce CXCL13. We have also reported that tumor-reactive CD8^+^ T cells produce CXCL13, and the frequency of those cells among the DP CD8^+^ cells positively correlated with the frequency of CXCL13^+^ DP CD4^+^ cells in both HNSCC and CRC (Supplemental Figure 2, A and B). Since tumor-reactive CD4^+^ and CD8^+^ T cells both secrete CXCL13, it is likely that this chemokine plays a key role in controlling the recruitment of immune cells to the TME as well as in the spatial organization of the immune cell infiltrate within the tumor. Future studies in mouse tumor models using selective knockout or neutralization of CXCL13 might help clarify the role of CXCL13 and potentially other chemokines in the antitumor response.

Our antigen reactivity analyses revealed that both DP CD4^+^ TILs and DP CD8^+^ TILs could recognize the HPV16 E6 protein. However, when dissecting the responses with 37 individual overlapping peptides, we found that DP CD4^+^ Th TILs had a broader response, recognizing several peptides of HPV16 E6 compared with DP CD8^+^ TILs (in the 3 patients analyzed). Furthermore, the regions of E6 recognized by DP CD4^+^ and DP CD8^+^ cells did not overlap. Similarly, our analyses of T cell reactivity against tumor-specific neoantigens have shown that DP CD4^+^ and CD8^+^ T cells recognized different peptides. These results are consistent with a recent study by Parkhurst et al. (30), in which the authors analyzed neoantigen reactivity in 75 patients with different gastrointestinal malignancies. In those 75 patients, the presence of both neoantigen-reactive CD4^+^ and CD8^+^ T cells was detected in 23 patients. Among those 23 patients, the nonsynonymous somatic mutations recognized by CD4^+^ and CD8^+^ T cells were distinct in all but 2 patients. Altogether, both studies imply that CD4^+^ and CD8^+^ T cell responses in tumors are complementary and together might overcome tumor cell heterogeneity and allow for broader tumor cell recognition by T cells. In a recent publication using a mouse methylcholanthrene-induced sarcoma model, in which the mutations recognized by CD4^+^ and CD8^+^ T cells were distinct (17), coexpression of those mutations in the same tumor cells was required for tumor rejection by immunotherapy. Hence, it might be important to determine in human tumor samples whether the nonsynonymous mutations recognized by CD4^+^ and CD8^+^ TILs are coexpressed in the same cell in patients with HNSCC and in those with CRC and whether coexpression has an impact on the efficacy of the antitumor immune response in these patients.

In conclusion, we have shown that among the CD4^+^ Th TILs, cells coexpressing PD-1 and ICOS were enriched for tumor reactivity. Understanding the cellular interactions between these cells and the other immune cells infiltrating the tumor might provide new strategies to treat patients with cancer. Furthermore, isolation of the DP PD-1^+^ICOS^+^ cell population could be used to enrich for tumor-reactive CD4^+^ Th cells for adoptive T cell therapy approaches.

## Methods

### Patient samples.

Peripheral blood, metastatic lymph nodes, and tumor samples were obtained from individuals with HNSCC or MSS colon cancers. At the time of sample collection, patients were not undergoing therapy. Previously, they had undergone a range of therapies, including chemotherapy, radiotherapy, surgery and immunotherapy, or none of the above.

PBMCs were purified from whole blood over a Ficoll-Paque PLUS (GE Healthcare) gradient and cryopreserved prior to analysis. Tumor specimens were prepared as follows: under sterile conditions, specimens were cut into small pieces and digested in RPMI-1640 medium in the presence of hyaluronidase at 0.5 mg/mL (MilliporeSigma, H6254), collagenase at 1 mg/mL (MilliporeSigma, C5138), and DNase at 30 U/mL (Roche, 04536282001) as well as human serum albumin (MP Biomedicals, catalog IC08823051) at a 1.5% final concentration. Cells were digested for 1 hour at room temperature (RT) under agitation with a magnetic stir bar. Cell suspensions were filtered through a 70 μm filter. In some cases, TILs were enriched as described above by Ficoll-Paque PLUS (GE Healthcare, catalog 17144003) density centrifugation. Tumor single-cell suspensions were cryopreserved until further analysis.

### Antibodies and flow cytometry.

All fluorescence-labeled antibodies used in this study are listed in Supplemental Table 2. After thawing, cryopreserved tumor digests and PBMCs were incubated with fixable live/dead dye to distinguish viable cells (BioLegend, Zombie Yellow Fixable Viability Kit, catalog 423104) and then stained with a combination of antibodies. Cell-surface staining was performed in FACS buffer (PBS supplemented with 1% FBS and 0.01% NaN3). Intracellular staining was performed using the Fix/Perm kit from eBioscience according to the manufacturer’s instructions. Stained cells were acquired on BD LSR II and LSRFortessa flow cytometers. Data were analyzed with FlowJo software, version 10.7.0 (Treestar). The t-SNE analysis was performed on concatenated tumor-infiltrating total CD4^+^ cells (4000 cells/patient) isolated from 22 patients with HNSCC and 16 patients with CRC using the t-SNE plugin in FlowJo software.

### Cell sorting and T cell expansion.

Cryopreserved PBMCs and TILs were thawed and labeled for sorting. In some cases, tumor samples were enriched for immune cells using the T Cell Enrichment Kit from STEMCELL Technologies or CD45 (TILs) MicroBeads from Miltenyi Biotec, following the manufacturers’ protocols. For TIL enrichment using the T Cell Enrichment Kit, EpCAM beads (STEMCELL Technologies) were added to the antibody cocktail. The enriched fractions were then labeled, and cell populations of interest were enriched via cell sorting to 99% purity on a BD FACSAria II. From PBMCs, naive CD4^+^ T cells were sorted as CD45^+^CD4^+^CD8^−^CD45RA^+^CCR7^+^CD127^+^CD25^−^ and memory CD4^+^ Th cells as CD45^+^CD4^+^CD8^−^CD45RA^−^CCR7^+/−^CD127^+^CD25^−^. From TILs, CD4^+^ Th subsets were sorted as CD45^+^CD4^+^CD8^−^CD45RA^−^CCR7^+/−^CD127^+/−^CD25^lo^PD-1^−^ICOS^−^ (DN CD4^+^), PD-1^+^ICOS^−^ (SP CD4^+^), and PD-1^+^ICOS^+^ (DP CD4^+^). CD8 subsets from TILs were sorted as CD45^+^CD4^−^CD8^+^CD45RA^–^CCR7^+/−^CD39^−^CD103^−^ (DN CD8^+^), CD39^−^CD103^+^ (SP CD8^+^), and CD39^+^CD103^+^ (DP CD8^+^). Sorted T cells (1000–2500 cells/well) were stimulated polyclonally in a 96-well, round-bottomed plate (Corning/Costar) with 1 μg/mL phytohemagglutinin (PHA) (Remel, catalog R30852801) in the presence of irradiated (50 Gy) allogeneic feeder cells (200,000 PBMCs per well) and 500–1000 IU/mL recombinant human IL-2 (Proleukin, Prometheus) for CD4^+^ T cells or 10 ng/mL recombinant human IL-15 (BioLegend, catalog 570304) for CD8^+^ T cells. Cells were cultured in complete RPMI-1640, supplemented with 2 mmol/L l-glutamine (Gibco, Thermo Fisher Scientific, catalog 25030081), 1% (vol/vol) nonessential amino acids (Gibco, Thermo Fisher Scientific, catalog 11140050), 1% (vol/vol) sodium pyruvate (Gibco, Thermo Fisher Scientific, catalog 11360070), penicillin (50 U/mL) plus streptomycin (50 μg/mL) (Gibco, Thermo Fisher Scientific, catalog 15140122), 10 μg/mL gentamicin (Thermo Fisher Scientific, catalog 15750-060), and 10% pooled human serum (in-house preparation). T cell lines were split when the wells reached confluence, and T cell lines were maintained in complete medium with IL-2 or IL-15 until analysis or cryopreserved in liquid nitrogen.

For TCR-Seq analysis, following cell-surface staining, cells were fixed with a 1:20 diluted fixation/permeabilization buffer (eBioscience) for 45 minutes, followed by 1 wash with FACS buffer and 1 wash with permeabilization buffer (eBioscience) according to the manufacturer’s instructions. The fixation/permeabilization buffer was titrated to 1:20 rather than 1:4 to minimize DNA damage by paraformaldehyde without hampering FOXP3 staining (31). Sorted cells were CD45^+^CD3^+^CD4^+^CD8^−^FOXP3^−^CD45RA^−^CCR7^+/−^PD-1^−^ICOS^−^ (DN), PD-1^+^ICOS^−^ (SP), and PD-1^+^ICOS^+^ (DP), and DNA extraction was performed.

### B cell expansion.

Autologous PBMCs were thawed, and CD19 Microbeads (Miltenyi Biotec, catalog 103-050-301) were used to isolate B cells. On day zero, 25,000 isolated B cells were seeded into 75 cm^2^ cell culture flasks (Greiner Bio-One, catalog 658175) in the presence of irradiated (60 Gy) 3T3-CD40 ligand cells (obtained in-house) in complete RPMI supplemented with 5% Human AB Serum (Valley Biomedical, catalog HP1022) and 10 ng/mL recombinant human IL-4 (BioLegend, catalog 574004). On days 4 and 6, fresh medium (11 mL) plus IL-4 (10 ng/mL) was added to the flasks. On day 8, B cells were harvested and restimulated (780,000 B cells/flask) in the presence of irradiated 3T3-CD40 ligand cells and 10 ng/mL IL-4. On days 10 and 12, fresh medium (11 mL) plus IL-4 (10 ng/mL) were added. On day 13 or 14, expanded B cells were counted and cryopreserved until further analysis.

### DNA preparation and TCR-Seq.

Deep sequencing of the variable V-J or V-D-J regions of TCR genes was performed on genomic DNA of sorted T cell populations. DNA was extracted from ex vivo–sorted CD4^+^ T cell subsets (DNeasy Blood and Tissue Kit, QIAGEN). The TCRβ CDR3 regions were sequenced and mapped using the human hsTCRB Sequencing kit (ImmunoSEQ, Adaptive Biotech). Samples were sequenced using a MiSEQ sequencer (Illumina). Coverage per sample was greater than 10×. Only data from productive rearrangements were extracted from the ImmunoSEQ Analyzer platform for further analysis. The Circos plots were created with the circlize R package (Gu, *Bioinformatics* 2014) and depict TCR repertoire similarity between different populations of cells. The ribbons connect a highlighted cell population with other populations that contain shared productive nucleotide sequences. Connections between the nonhighlighted cell populations are not depicted. The width of the ribbon at each end reflects the proportion of total sequences in that population that are shared with the connected population. To compare the TCR Vβ overlap (or similarity) of 2 given cell populations, we used the Morisita overlap index. For the comparison of shared clones between subsets, we analyzed the top 30 clones in each subset.

### mIHC.

Formalin-fixed, paraffin-embedded (FFPE) blocks were cut into serial sections of 4 μm thickness and placed onto Superfrost Ultra Plus Adhesion slides (Thermo Fisher Scientific). Tissue slides were stained manually. All sections were deparaffinized, rehydrated, and subjected to heat-induced epitope retrieval (Akoya Biosciences). Samples were evaluated using the following multiplex immunofluorescence staining panel: anti-FOXP3 (Thermo Fisher Scientific, 236A/E7; no. 14-4777-82); anti-CD8 (Abcam, SP16; no. ab101500); anti-CD3 (Abcam, SP7; no. ab16669); anti-ICOS (Cell Signaling Technology, D1K2T, no. 89601S); anti–MHC class II (BD Pharmingen, Tu39; no. 555556); and anti-cytokeratin (BioLegend, C-11; no. 628602). Antigen-antibody binding was visualized with TSA-Opal reagents (Akoya Biosciences). Detailed information regarding antibody dilution, antibody/TSA-Opal reagents combination, and antigen retrieval buffers is provided in Supplemental Table 3. Tissue slides were incubated with DAPI as a counterstain and coverslipped with VectaShield mounting media (Vector Laboratories).

### mIHC image acquisition and analysis.

Digital images were acquired with a Vectra 3.0 Automated Quantitative Pathology Imaging System (Akoya Biosciences). A ×10 objective lens was used for whole-slide scans to identify the regions of interest (ROIs). A ×20 objective lens was used for the multispectral images (MSIs). Seven to 15 ROIs were selected from each tissue sample for mIHC analysis. ROIs that contained at least 30% tumor cells were selected. Tissue segmentation was performed according to cytokeratin and DAPI staining. Cell segmentation and phenotyping of individual cells were performed according to individual markers and the presence of DAPI using Inform 2.4 software (Akoya Biosciences). For tumor versus stroma analysis, the stamp size for the ROIs was 670 × 502 μm^2^. For the nearest-neighbor analysis, the stamp size for the ROIs was 1340 × 1004 μm^2^. Consecutive tissue slides for each case were stained using the conventional H&E method to ensure the presence of tumor cells and to evaluate the fixation quality. Tissue slides were digitally scanned with the Leica SCN400F platform at ×20 and magnified at ×200 to ×400 for immune infiltration evaluation.

### WES and RNA-Seq.

Genomic DNA and total RNA were purified from 5 μm FFPE tumor sections on an automated QiaCube instrument using DNA/RNA AllPrep reagents (QIAGEN) according to the manufacturer’s instructions. The corresponding normal DNA for germline exome testing was purified from the matching patient’s in vitro–expanded T cells or PBMCs, as described above. DNA and RNA were quantified using a Qubit fluorometer (Thermo Fisher Scientific). WES for tumor and germline specimens was performed on purified DNA as follows: DNA was prepared into tagged sequencing libraries using Kapa HyperPlus library preparation reagents (Roche), and exome hybrid capture was performed using the xGen Research Panel kit (Integrated DNA Technologies [IDT]). Captured library pools were normalized and loaded onto a HiSeq 4000 sequencer (Illumina) for next-generation sequencing. WES reads were aligned to the Genome Reference Consortium Human Build 37 (hg19) followed by GATK preprocessing. Four independent single nucleotide mutation callers (Varscan 2.3.6, SomaticSniper 1.0.5.0, Mutect 1.1.7, and Strelka 1.0.15) were used to call somatic, nonsynonymous single nucleotide variants, and 2 insertion-deletion (indel) callers (Strelka 1.0.15 and Varscan 2.3.6) were used to identify somatic, nonsynonymous indels. The identified mutations were filtered according to the following criteria: minimum coverage of 10 reads, greater than 5% variant allele frequency (VAF), and called by 2 or more callers. Somatic mutations that passed the filters were further annotated with the 1000 Genomes Project, Exome Aggregation Consortium (ExAC), and the Catalogue of Somatic Mutations in Cancer (COSMIC) databases using Annovar. SNPeff was used to predict variant functional effect. Every nonsynonymous mutation was used to build putative neoepitopes of 25 mer amino acid sequences. An mRNA sequencing library was also prepared from FFPE tissues using RNA Access Library Preparation reagents (Illumina) according to the manufacturer’s instructions. Libraries were pooled and sequenced at a depth of 25 to 50 million reads on a HiSeq 4000 sequencer (Illumina). RNA alignment was performed using STAR, followed by GATK preprocessing best practices workflow. Fragments per kb per million mapped reads (FPKM) values were calculated using Cufflinks. FPKM levels were used to assess the expression of candidate mutations.

### Peptide synthesis.

Potential 25 mer neoantigen sequences were synthesized by GenScript USA and subsequently tested for reactivity against patients’ CD4^+^ and CD8^+^ T cell subsets. Crude peptides were used for the initial in vitro screening. To validate reactivities, selected HPLC-purified mutant peptides and their WT counterparts were purchased from GenScript USA. To screen for recognition of HPV antigens, we used 2 overlapping peptide libraries, encoding for the full-length amino acid sequences of HPV16 E6 and E7 oncoproteins (159 and 99 amino acids in length, respectively). The 15 mer peptides in each pool overlapped by 11 amino acids. HPV16 E6 contained 37 peptides, and HPV16 E7 contained 22 peptides in each pool. Peptides were synthesized by GenScript USA. For the confirmation of neoantigen reactivity of the DP CD8^+^ TILs for patient 1 and patient 3, the potential 25 mer neoantigen sequences were converted into FASTA format and run through the NetMHCpan 4.0 Server (Technical University of Denmark). NetMHCpan generates 8 mer to 11 mer peptides from the 25 mer neoantigen sequences and predicts binding affinity to patient-specific MHC class I molecules. Peptides with a percentage rank below 2 were considered candidates for further evaluation. Those peptides were synthesized by GenScript USA and subsequently tested for reactivity against patients’ DP CD8^+^ TILs.

### Peptide pulsing.

B cells were thawed and plated at 100,000 cells/well in complete RPMI supplemented with 10 ng/mL IL-4 in a 96-well, round-bottomed plate. Long peptides (25 mers, GenScript USA) were added at 5 μg/mL (or the indicated concentrations for titrations), and cells were incubated overnight at 37°C with 5% CO_2_. The following day, B cells were washed twice prior to coculturing with T cells. For initial screening, long peptides were pooled (9–11 peptides/pool). In subsequent screenings, reactive pools were deconvoluted, and individual peptides were used at 5 μg/mL. For reactivity to common viruses, the following Peptivator peptide pool reagents from Miltenyi Biotec were used: Peptivator CMV pp65, Peptivator Influenza A (H1N1) HA, Peptivator Influenza A (H1N1) NP, Peptivator EBV EBNA-1, and Peptivator EBV BZLF1. In some cases, autologous monocytes were used as APCs. Monocytes were isolated from cryopreserved, autologous PBMCs using CD14 MicroBeads (Miltenyi Biotec), and 10,000–25,000 monocytes/well were pulsed separately with HPV peptide pools for 2 to 4 hours.

### Electroporation of HPV RNA.

Cryopreserved autologous PBMCs or autologous expanded memory CD8^+^ T cells were thawed, labeled with Cell Proliferation Dye eFluor 450 (eBioscience, catalog 65084285), and then washed and resuspended in Opti-MEM (Life Technologies, Thermo Fisher Scientific, catalog 31985062) at 10 × 10^6^ to 30 × 10^6^ cells/mL. HPV RNA (4 μg) was added to a 2 mm gap electroporation cuvette (BTX 45-0125, Cole-Parmer), followed by 50 μL autologous cells. Electroporation was performed at 250 V for 5 ms for 1 pulse, using a BTX ECM 830 Square Wave Electroporation System (Harvard Bioscience). Electroporated cells were incubated in complete medium at 1.3 × 10^6^ to 2.5 × 10^6^ cells/mL for 4 hours before coculturing with CD8^+^ T cell subsets.

### Coculture assays with IFN-γ ELISPOT and flow cytometry to assess T cell activation.

The reactivity to predicted neoepitopes and HPV antigens was assessed via a paired ELISPOT assay and detection of activation markers (OX40, 4-1BB) by flow cytometry. CD4^+^ and CD8^+^ T cells were thawed and rested for 2 days at 37°C in complete medium with IL-2 (500 IU/mL) or IL-15 (10 ng/mL), respectively. In coculture experiments, 100,000 T cells/well were used, and all cocultures were performed in the absence of exogenous cytokines. For coculturing with B cells or monocytes and CD4^+^ T cells, SEB (Toxin Technology, catalog BT-202, 1 μg/mL) was used as a positive control. For coculturing with CD8^+^ T cells, plate-bound anti-CD3 antibody (OKT3, BioLegend, 1 μg/mL) was used as a positive control. The expression of HPV16 transcripts for E6 and E7 was confirmed by RNAscope (Advanced Cell Diagnostics [ACD]) (Supplemental Figure 4).

For IFN-γ ELISPOT assays, ELIIP plates (MilliporeSigma, MAIPSWU10) were activated with 50 μL per well of 70% ethanol for 2 minutes, washed 3 times with PBS, and then coated with 50 μL of 10 μg/mL IFN-γ capture antibody (Mabtech, clone 1-D1K) and incubated overnight in the refrigerator. For OKT3 controls, wells were coated with a mixture of IFN-γ capture antibody (10 μg/mL) and OKT3 (1 μg/mL). Prior to coculturing, the plates were washed once with PBS, followed by blocking with complete RPMI media for at least 1 hour at RT. Pulsed APCs and T cells were added to the ELISPOT plate and incubated at 37°C. After 20 to 24 hours of coculturing, cells were harvested from the ELISPOT plate into a standard 96-well, round-bottomed plate for flow cytometric analysis (as described below), and then the ELISPOT plates were washed with PBS plus 0.05% Tween-20 (PBST) and incubated for 2 hours at RT with 100 μL/well of a 0.22 μm filtered 1 μg/mL biotinylated anti–human IFN-γ detection antibody solution (Mabtech, clone: 7-B6-1). The plate was then washed with PBS-T, followed by a 1-hour incubation with 100 μL/well streptavidin-ALP (Mabtech). The plate was washed with PBS followed by development with 100 μL/well of 0.45 μm filtered BCIP/NBT substrate solution (KPL). The reaction was stopped by rinsing thoroughly with cold tap water. ELISPOT plates were scanned and counted using an ImmunoSpot plate reader and the associated software (Cellular Technology).

Expression of the T cell activation markers OX40 (CD4) and 4-1BB (CD8) was assessed by flow cytometry after 20 to 24 hours of coculturing. Briefly, cells that were harvested from the ELISPOT plate were washed with PBS and stained with a fixable live/dead dye to distinguish viable cells (eBioscience, Fixable Viability Dye eFluor 780) for 8 minutes at 4°C in the dark. Cells were washed twice and then stained with the appropriate antibodies diluted in FACS buffer for 20 minutes at 4°C in the dark. Cells were washed twice with FACS buffer prior to acquisition on a BD LSR II flow cytometer. Data were analyzed with FlowJo software, version 10.7.0 (Tree Star).

### RNA isolation and qPCR.

Total RNA from sorted T cell subsets was extracted using the NucleoSpin RNA XS from Macherey-Nagel (catalog 740902.50), according to the manufacturer’s instructions (no RNA carrier was used). cDNAs were synthesized with the RevertAid RT Kit (Thermo Fisher Scientific, catalog K1691) using oligo(dT) primers (Thermo Fisher Scientific, catalog S0132). Transcripts were quantified by real-time qPCR on an ABI 7500 Fast Real-Time PCR system (Applied Biosystems) using the SYBR Green qPCR Master kit from Thermo Fisher Scientific (catalog K02520). For each sample, mRNA abundance was normalized to the amount of RPS18 RNA and expressed as AU using the 2^–ΔCt^ method. The primers used are listed in Supplemental Table 4.

### Data availability.

TCR-Seq data were deposited in the ImmuneACCESS database (Adaptive Biotechnologies), available at DOI:10.21417/RD2022JCI (https://clients.adaptivebiotech.com/pub/duhen-2022-jci). The somatic mutations carried by the 4 patients analyzed, as well as the sequences of the predicted peptides are listed in Supplemental Table 2. The underlying WES data are not publicly available because of HIPAA protection of the patients’ germline sequencing data. We do not have patient consent to release read-level RNA-Seq data containing private/rare variants. Any inquiries to access these data (including RNA-Seq data) should be directed to eacri.bioinformatics@providence.org, and we will grant access to the deidentified data sets for research purposes, with a data transfer agreement.

### Statistics.

Statistical significance between groups was determined by 1-way ANOVA with Tukey’s correction or by unpaired 2-tailed *t* test. For each test, a *P* value of less than 0.05 was considered statistically significant. Analyses were performed with GraphPad Prism 9 (GraphPad Software).

### Study approval.

All surgical tumor samples and blood samples used in this study were obtained from individuals treated at the Providence Cancer Institute. All patients provided written informed consent. This study was approved b+y the Providence Portland Medical Center IRB (IRB protocol no. 06-108A) and was conducted in accordance with the ethics standards established by the Declaration of Helsinki.

## Author contributions

RD designed research, conducted experiments, analyzed data, and wrote the manuscript. OF conducted experiments, analyzed data, and revised the manuscript. KAS performed and analyzed the mIHC experiments and processed tumor samples. AKF conducted and analyzed tumor reactivity experiments. MB processed tumor samples and performed RNAScope experiments for HPV16 E6 and E7. VR and BB developed the neoantigen pipeline and analyzed WES and RNA-Seq samples. DR developed the Circos plot analysis tool and the cellular spatial relationship map tool. ET provided the plasmid construct for HPV reactivity and revised the manuscript. ADW designed and supervised research and wrote the manuscript. TD designed and supervised research, conducted experiments, analyzed the data, and wrote the manuscript.

## Supplementary Material

Supplemental data

Supplemental table 1

Supplemental table 2

Supplemental table 3

Supplemental table 4

## Version 1 04/19/2022

In-Press Preview

## Version 2 06/15/2022

Electronic publication

## References

[1] Le DT (2015). PD-1 blockade in tumors with mismatch-repair deficiency. N Engl J Med.

[2] Le DT (2017). Mismatch repair deficiency predicts response of solid tumors to PD-1 blockade. Science.

[3] Overman MJ (2017). Nivolumab in patients with metastatic DNA mismatch repair-deficient or microsatellite instability-high colorectal cancer (CheckMate 142): an open-label, multicentre, phase 2 study. Lancet Oncol.

[4] Addeo R (2019). CheckMate 141 trial: all that glitters is not gold. Expert Opin Biol Ther.

[5] Galon J (2006). Type, density, and location of immune cells within human colorectal tumors predict clinical outcome. Science.

[6] Sato E (2005). Intraepithelial CD8^+^ tumor-infiltrating lymphocytes and a high CD8^+^/regulatory T cell ratio are associated with favorable prognosis in ovarian cancer. Proc Natl Acad Sci U S A.

[7] de Ruiter EJ (2017). The prognostic role of tumor infiltrating T-lymphocytes in squamous cell carcinoma of the head and neck: A systematic review and meta-analysis. Oncoimmunology.

[8] Duhen T (2018). Co-expression of CD39 and CD103 identifies tumor-reactive CD8 T cells in human solid tumors. Nat Commun.

[9] Simoni Y (2018). Bystander CD8^+^ T cells are abundant and phenotypically distinct in human tumour infiltrates. Nature.

[10] Thommen DS (2018). A transcriptionally and functionally distinct PD-1^+^ CD8^+^ T cell pool with predictive potential in non-small-cell lung cancer treated with PD-1 blockade. Nat Med.

[11] Rajamanickam V (2021). Robust antitumor immunity in a patient with metastatic colorectal cancer treated with cytotoxic regimens. Cancer Immunol Res.

[12] van den Bulk J (2019). Neoantigen-specific immunity in low mutation burden colorectal cancers of the consensus molecular subtype 4. Genome Med.

[13] Chaplin DD (2010). Overview of the immune response. J Allergy Clin Immunol.

[14] Laidlaw BJ (2016). The multifaceted role of CD4(+) T cells in CD8(+) T cell memory. Nat Rev Immunol.

[15] Linnemann C (2015). High-throughput epitope discovery reveals frequent recognition of neo-antigens by CD4^+^ T cells in human melanoma. Nat Med.

[16] Yossef R (2018). Enhanced detection of neoantigen-reactive T cells targeting unique and shared oncogenes for personalized cancer immunotherapy. JCI Insight.

[17] Alspach E (2019). MHC-II neoantigens shape tumour immunity and response to immunotherapy. Nature.

[18] Tran E (2014). Cancer immunotherapy based on mutation-specific CD4^+^ T cells in a patient with epithelial cancer. Science.

[19] Montler R (2016). OX40, PD-1 and CTLA-4 are selectively expressed on tumor-infiltrating T cells in head and neck cancer. Clin Transl Immunology.

[20] Mackay LK (2015). Cutting edge: CD69 interference with sphingosine-1-phosphate receptor function regulates peripheral T cell retention. J Immunol.

[21] Bromley SK (2005). Chemokine receptor CCR7 guides T cell exit from peripheral tissues and entry into afferent lymphatics. Nat Immunol.

[22] Debes GF (2005). Chemokine receptor CCR7 required for T lymphocyte exit from peripheral tissues. Nat Immunol.

[23] Brown MN (2010). Chemoattractant receptors and lymphocyte egress from extralymphoid tissue: changing requirements during the course of inflammation. J Immunol.

[24] Crotty S (2019). T follicular helper cell biology: a decade of discovery and diseases. Immunity.

[25] Jeon S, Lambert PF (1995). Integration of human papillomavirus type 16 DNA into the human genome leads to increased stability of E6 and E7 mRNAs: implications for cervical carcinogenesis. Proc Natl Acad Sci U S A.

[26] Kortekaas KE (2020). CD39 identifies the CD4^+^ tumor-specific T cell population in human cancer. Cancer Immunol Res.

[27] Scheper W (2019). Low and variable tumor reactivity of the intratumoral TCR repertoire in human cancers. Nat Med.

[28] Gu-Trantien C (2013). CD4^+^ follicular helper T cell infiltration predicts breast cancer survival. J Clin Invest.

[29] Gu-Trantien C (2017). CXCL13-producing TFH cells link immune suppression and adaptive memory in human breast cancer. JCI Insight.

[30] Parkhurst MR (2019). Unique neoantigens arise from somatic mutations in patients with gastrointestinal cancers. Cancer Discov.

[31] Ahmadzadeh M (2019). Tumor-infiltrating human CD4^+^ regulatory T cells display a distinct TCR repertoire and exhibit tumor and neoantigen reactivity. Sci Immunol.

