# Supplementary material for: PD-1 and ICOS coexpression identifies tumor-reactive CD4+ T cells in human solid tumors
Source: J Clin Invest. 2022 Jun 15;132(12):e156821. doi: 10.1172/JCI156821 (PMC9197519; doi:10.1172/JCI156821)
Supplement: Supplemental data [file jci-132-156821-s034.pdf]

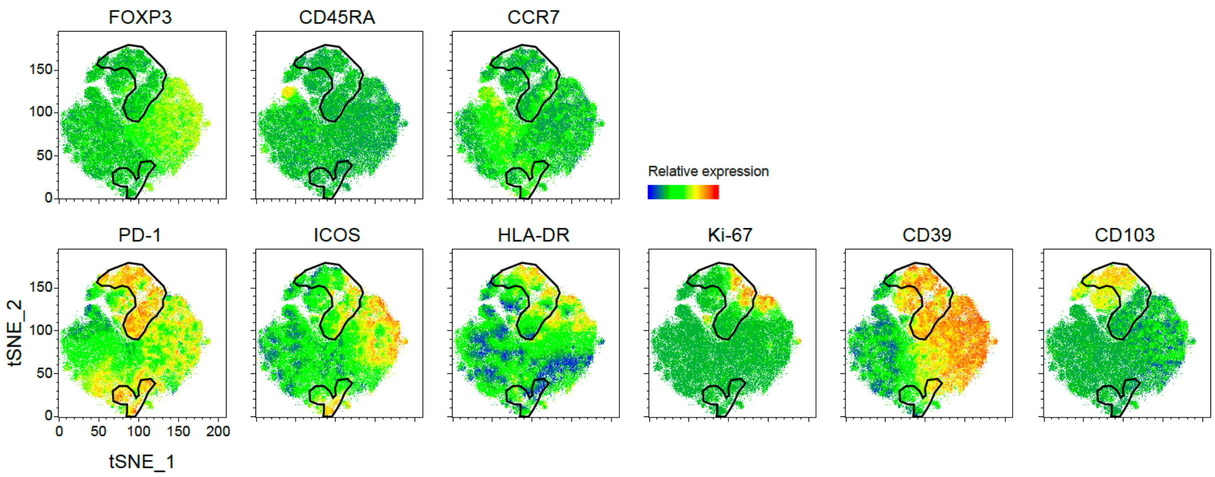

Supplemental Figure 1. PD-1 and ICOS identify different subsets of CD4 TIL

t-SNE analysis of tumor-infiltrating CD3+CD4+ T cells isolated from 16 patients with CRC. The gate identifies PD-1+ cells. The gate is applied to all plots showing expression levels of ICOS, HLA-DR, Ki-67, CD39 and CD103.

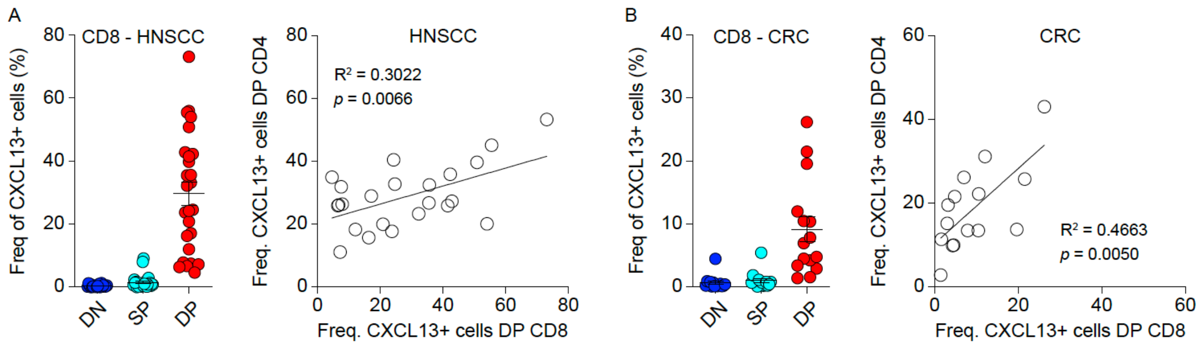

Supplemental Figure 2. CXCL13 production by DP CD4 Th TIL and CD8 TIL.

A) Frequency of CXCL13-producing cells in each CD8 TIL subset in HNSCC patients (n= 28) (left). Correlation between the frequency of CXCL13-producing cells in DP CD8 and DP CD4 TILs (n= 23) (right). **B**) Frequency of CXCL13-producing cells in each CD8 TIL subset in CRC patients (n= 25) (left). Correlation between the frequency of CXCL13-producing cells in DP CD8 and DP CD4 TIL (n= 15) (right).

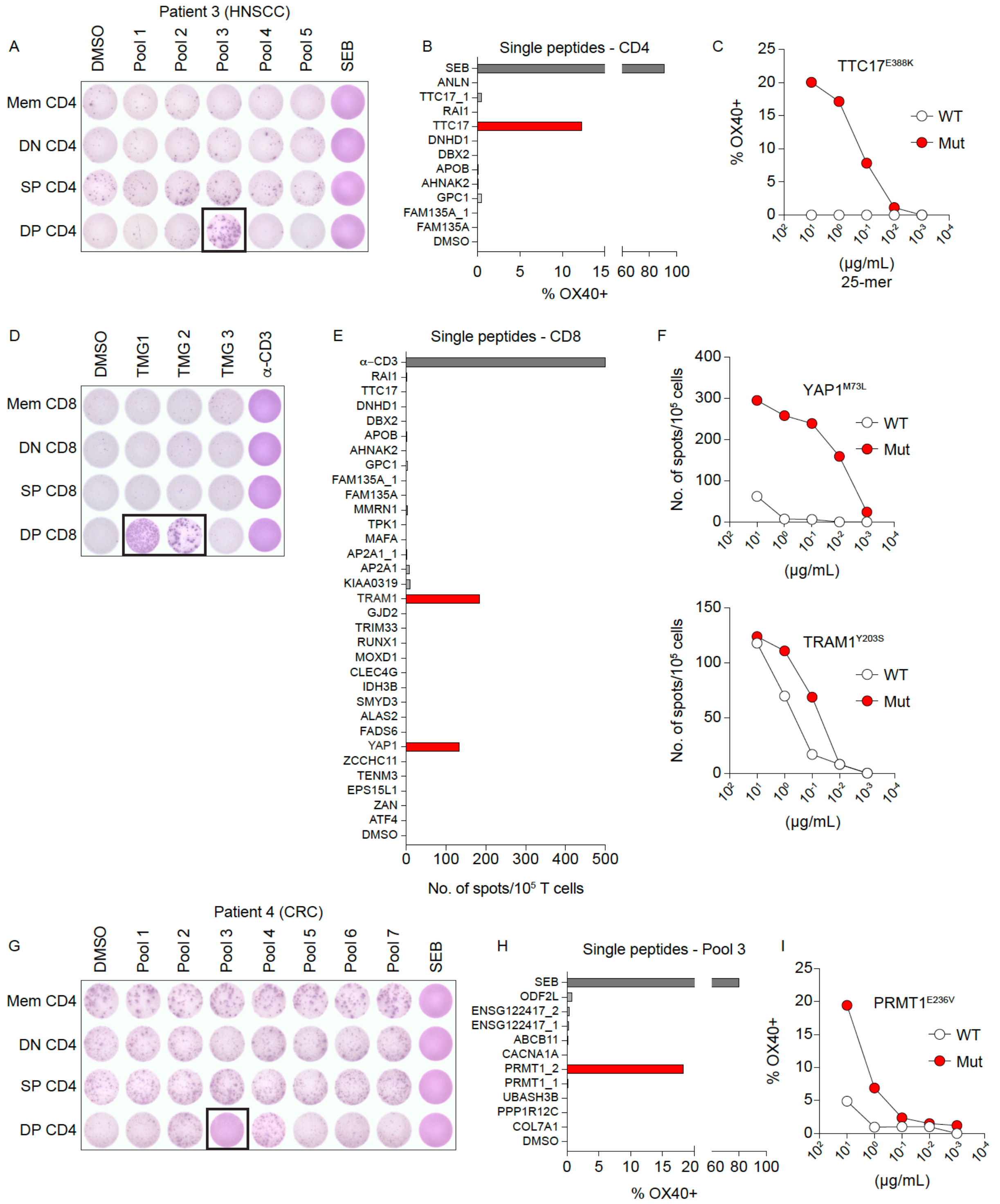

Supplemental Figure 3. DP CD4 Th TIL recognize tumor-specific neoantigens.

A) In-vitro expanded CD4 T cell subsets (DN, SP and DP) from patient 3 were cocultured with autologous B cells pulsed with DMSO or the indicated peptide pools containing the 25-mers with the putative mutations identified by WES. T cell reactivity was measured by IFN-γ ELISPOT assay. **B**) OX40 up-regulation by DP CD4 Th TIL after coculture with B cells pulsed with individual 25-mers from peptide pool #3. The mutation recognized is highlighted in red. **C**) OX40 up-regulation by DP

CD4 Th TIL after coculture with B cells pulsed with decreasing concentrations of wt or mutated (mut) TTC17^{E388K} 25-mers. D) In-vitro expanded CD8 T cell subsets (DN, SP and DP) from patient 3 were cocultured with autologous memory CD8 T cells electroporated with *in-vitro* transcribed RNA encoding for TMGs. T cell reactivity was measured by IFN-γ ELISPOT assay. **E**) Reactivity of DP CD8 TILs to B cells pulsed with individual 25-mers corresponding to mutations present in TMG #1 and #2 measured by IFN-γ ELISPOT assay. The mutations recognized are highlighted in red. **F**) 4-1BB up-regulation by DP CD8 TIL after coculture with B cells pulsed with decreasing concentrations of wt or mut YAP1^{M73L} 10-mers and wt or mut TRAM1^{Y203S} 25-mers. G) In-vitro expanded CD4 T cell subsets (DN, SP and DP) from patient 4 were cocultured with autologous B cells pulsed with DMSO or the indicated peptide pools containing the 25-mers with the putative mutations identified by WES. T cell reactivity was measured by IFN-γ ELISPOT assay. **H**) OX40 up-regulation by DP CD4 TIL after coculture with B cells pulsed with individual 25-mers from peptide pool #3. The mutation recognized is highlighted in red. **I**) OX40 up-regulation by DP CD4 TIL after coculture with B cells pulsed with decreasing concentrations of wt or mutated (mut) PRMT1^{E236V} 25-mers.

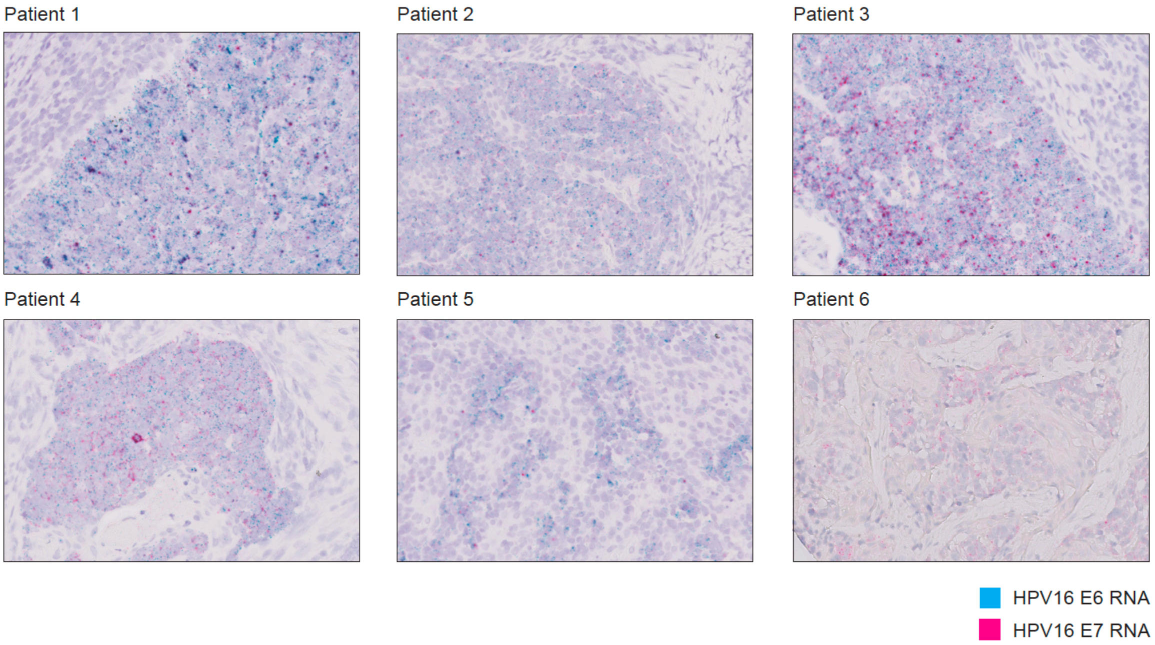

Supplemental Figure 4. Detection of HPV16 *E6* and *E7* transcripts by RNAscope.

RNAscope in situ hybridization of FFPE tissue sections from 6 HPV+ HNSCC patients, stained for the presence of HPV16 *E6* and *E7* RNA transcripts (cyan depicts HPV16 *E6* RNA, magenta depicts HPV16 *E7* RNA).
